# Sacituzumab Govitecan in Triple Negative Breast Cancer: A Systematic Review of Clinical Trials

**DOI:** 10.3390/cancers16213622

**Published:** 2024-10-27

**Authors:** Marcelino Pérez-Bermejo, Mónica Caballero-Pascual, María Ester Legidos-García, Miriam Martínez-Peris, Jorge Casaña-Mohedo, Francisco Llorca-Colomer, Ignacio Ventura, Francisco Tomás-Aguirre, Adalberto Asins-Cubells, María Teresa Murillo-Llorente

**Affiliations:** 1 SONEV Research Group, Faculty of Medicine and Health Sciences, Catholic University of Valencia, C/Quevedo Nº 2, 46001 Valencia, Spain; ester.legidos@ucv.es (M.E.L.-G.); miriam.martinez@ucv.es (M.M.-P.); jorge.casana@ucv.es (J.C.-M.); llorfran@mail.ucv.es (F.L.-C.); paco.tomas@ucv.es (F.T.-A.); mt.murillo@ucv.es (M.T.M.-L.); 2 School of Medicine and Health Sciences, Catholic University of Valencia, C/Quevedo Nº 2, 46001 Valencia, Spain; monica.caballero@mail.ucv.es; 3 Molecular and Mitochondrial Medicine Research Group, School of Medicine and Health Sciences, Catholic University of Valencia, C/Quevedo Nº 2, 46001 Valencia, Spain; ignacio.ventura@ucv.es; 4 Centro de Salud de L’Eliana, Departamento Arnau de Vilanova-Lliria, C/Rosales, 23, L’Eliana, 46183 Valencia, Spain; asins_ada@gva.es

**Keywords:** triple-negative breast cancer, sacituzumab govitecan, chemotherapy, antibody–drug conjugate, Trop-2 antigen, bystander effect, UGT1A1

## Abstract

Background/Objectives: Triple-negative breast cancer is difficult to treat due to the absence of hormone receptors and Her2neu. Sacituzumab govitecan is a new therapeutic approach that uses an antibody directed against the Trop-2 antigen present in solid epithelial tumors, linked to the active metabolite SN-38, similar to irinotecan, to specifically target cancer cells while minimizing damage to healthy cells. The objective of the present review was to evaluate the efficacy and safety of sacituzumab govitecan as a single treatment in patients with triple-negative breast cancer and to compare its results with the standard conventional chemotherapy regimen currently used in this disease. Methods: A systematic review of randomized clinical trials of sacituzumab govitecan was performed. The search was performed in Medline (PubMed), Web of Science, and Cochrane from September 2022 to January 2024. Results: Thirty-eight articles are included and evaluated according to inclusion and exclusion criteria corresponding to the two most relevant clinical trials, including specific analyses of cohorts and subgroup study arms within these trials. Data from more recent clinical trials are also reviewed. Conclusions: The efficacy results showed a significantly greater clinical benefit with sacituzumab govitecan compared to standard chemotherapy in patients with triple-negative breast cancer. This drug will become a treatment of substantial impact in future treatment guidelines for this type of cancer.

## Simple Summary

Sacituzumab govitecan is a promising treatment option for patients with triple-negative breast cancer, a type of cancer that is difficult to treat due to the absence of hormone receptors, specifically human epidermal growth factor receptor 2. This study is designed to evaluate the efficacy and safety of sacituzumab govitecan, which targets the trophoblast cell surface antigen 2 found on tumor cells, compared to standard chemotherapy. Based on a systematic review of clinical trials, the results indicate that sacituzumab govitecan significantly improves clinical outcomes, providing greater therapeutic benefit with fewer side effects compared to conventional treatments. These findings may influence future treatment guidelines for this aggressive cancer and provide a new approach to its management.

## 1. Introduction

Breast cancer (BC) is the most commonly diagnosed solid cancer among women [1,2]. It represents the second leading cause of female death [1,3] and the leading cause of female cancer mortality today. These data position breast cancer as a serious public health problem worldwide [2].

When a breast lump suggestive of malignancy is discovered, we do a biopsy to determine the nature of the mass. This procedure includes a subsequent anatomopathological and immunohistochemical (IHC) examination of the primary tumor, the results of which provide prognostic and predictive information about the disease according to the expression of membrane receptors such as Her2neu and nuclear receptors such as estrogen (ER) and progesterone (PR) receptors [3,4].

According to the combinations of these receptors, four intrinsic BC subtypes are identified according to the IHC phenotype of the primary tumor, which provides critical information for the treatment of each subtype [1,3,5].

Triple-negative breast cancer (TNBC) accounts for approximately 10–24% of all breast neoplasms [3,4,6,7,8]. This cancer subtype is characterized by the absence of molecular targets [1,4,5,9], specifically hormone membrane receptors and Her2neu, which, in contrast to the Luminal A/B and Her2neu subtypes, greatly complicates the selection of appropriate targeted therapy [1,2,3,4,5,6,7,8,9].

Recent work has shown that BC membrane receptors can change their presence or absence during tumor progression, which has a significant impact on treatment efficacy and patient survival [3,4,5,6,7,8,9]. Thus, a new concern arises for patients diagnosed with luminal A/B or Her2neu cancers, as they may become membrane receptor negative during tumor progression and develop secondary TNBC despite established therapies [10].

In addition, TNBC has a high proliferation rate, with Ki-67 over 80% [2]. The combination of receptor deficiency and high cell proliferation rate makes it the most aggressive subtype of BC [1,2,5,7], limiting effective therapeutic options [2] and resulting in an unfavorable prognosis [1,7], an increased risk of recurrence and distant metastasis [8], and a low survival rate [1,3,4,5,7,8].

Currently, the standard first-line treatment for TNBC is chemotherapy, although it has been associated with low tumor response rates, limited progression-free survival, and short overall survival [1,2,3,4]. Some patients show tumor responses to chemotherapy, particularly those with newly diagnosed or stage T1N0M0 TNBC, but outcomes are highly variable and there is a significant likelihood of cancer progression [1,6,7].

Given the challenging nature of TNBC and the lack of specific and effective therapeutic options, new approaches to cancer treatment have emerged, such as immune-molecule-based therapies [1,5,8]. These options include drug-conjugated monoclonal antibodies (DCAs), which are discussed in this review [11,12]. They consist of an antibody (Ab) capable of recognizing specific antigens (Ag) present or overexpressed on tumor membranes, combined with cytotoxic chemotherapeutic agents. Once Ab–Ag binding is established, the Ag undergoes endocytosis and internalizes the DCA into the tumor cells. After internalization, the cytotoxic drug is transported to lysosomes and released into the interior of the cell. Toxic cargoes induce apoptosis or growth inhibition [4,11,12,13,14,15,16,17]. The main advantage of this type of approach in TNBC is that since the Ab are bound to the cytotoxic agent, the drug is guaranteed to be released specifically into tumor cells, avoiding the systemic effects associated with chemotherapy and significantly reducing toxicity to healthy cells [14,15,16,17].

An example of an TDXD is trastuzumab, which targets Her2neu receptors overexpressed on the tumor membrane of the Her2neu tumor subtype and is currently the treatment of choice for this tumor type [11,12,14,15,16]. However, since TNBC lacks Her2neu receptors, trastuzumab cannot bind to tumors that do not express this Ag, making it an unsuitable ACA for the treatment of this cancer subtype, prompting the scientific community to identify new specific tumor markers on the TNBC surface, including the subject of our study, human trophoblast cell surface Ag 2 (Trop 2) [11,12,14,15,16].

### Sacituzumab Govitecan

**Figure 1:**
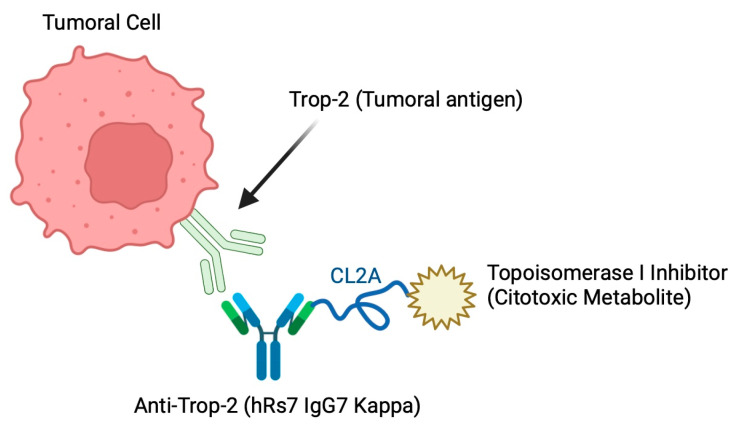
Structure of SG. Own elaboration based on [11,12,16,18,19,20,21,22,23,24].

The first two generations of ADCs were characterized by increased stability and toxicity issues due to suboptimal antibody–drug binding, which limited their efficacy and safety. An example of the first generation is gemtuzumab ozogamicin, while examples of the second generation include brentuximab vedotin, which improved drug stability and specificity. The present work focuses on the third-generation sacituzumab govitecan (SG), which incorporates more effective linkers and improved cytotoxic payloads, increasing precision and minimizing side effects. SG consists of three components: an Ag Trop 2 targeting Ac, termed anti-Trop 2 hRs7 (IgG7 kappa) (Trodelvy), a hydrolyzable linker and an active metabolite (SN-38), and a topoi-somerase I inhibitor (Figure 1) [11,12,16,18,19].

Ag Trop 2 is a transmembrane glycoprotein that acts as an intracellular calcium signal transducer. Notably, this Ag is 80–90% overexpressed on the surface of various solid tumors (BC, pancreatic, colon, prostate, and lung), including TNBC. However, its expression in healthy tissues is limited [12,16,18,19,20,21]. Furthermore, Trop 2 has been shown to be associated with cell proliferation, tumor growth, metastatic invasion, and regulation of mesenchymal–epithelial transcription. These properties make Trop 2 a very attractive therapeutic target in cancer [16,18,19,20,21,22]. The H-score of Ag Trop 2 has been used as a quantitative method to evaluate its immunohistochemical expression, categorized into three levels: low (0–100), intermediate (100–200), and high (200–300). This classification has been used in several studies related to Trop 2, both in TNBC and in other cancer subtypes such as early-stage luminal cancer. Although no clear consensus has been reached on the clinical and biological relevance of the variability in expression of this Ag in response to targeted therapies, it does not appear to have a direct relationship with tumor aggressiveness. Nevertheless, it remains an active field of research, as there is still no conclusive evidence about the functional and clinical implications of the presence of Trop 2 [25,26].

Anti-Trop 2 hRs7 binds via a proprietary hydrolyzable connector (CL2A) to the membrane-permeable cytotoxic cargo corresponding to 7–8 molecules of SN-38, which happens to be the active metabolite of the chemotherapeutic agent irinotecan. This is internalized into the cell and taken up by the lysosomes, which degrade the Ab, thus releasing the cytotoxic into the tumor cell microenvironment, and SN-38 proceeds to act as an inhibitor of topoisomerase I, causing DNA breakage and inducing cell apoptosis. This SN-38-associated DCA has been shown to be 1000 times more effective in cancer treatment than the chemotherapy irinotecan, despite having the same metabolite. This is due to the fact that its systemic administration has serious side effects, but its targeted release by SG allows to reduce them without losing the efficacy and potency of the metabolite itself on malignant cells [11,12,16,18,19,20,21,22].

SG exhibits a unique feature known as the “bystander effect,” which, together with drug specificity, adds another dimension to the efficacy and potency of Ab-bound SN-38 compared to the chemotherapeutic agent irinotecan. This specificity lies in the fact that SN-38, being cell membrane permeable, can diffuse to neighboring cancer cells after initially binding to a tumor cell overexpressing Trop 2. This is significant because some of these neighboring cells may lack Ag Trop 2 in their membranes, which would in principle make them inaccessible to SG. However, this effect allows apoptosis to be induced in cells that do not express Trop 2, further enhancing the efficacy of the drug [22,23,24,27].

SG allows for precise drug delivery into the tumor microenvironment, minimizing damage to healthy cells. This DCA specifically binds to Trop 2, internalizes, and then releases SN-38 in a targeted manner by cleaving the hydrolyzable connector. This mechanism of action suggests that SG could provide longer progression-free and overall survival in the treatment of TNBC compared to first-line standard chemotherapy in this cancer. This is due to its targeted action and its ability to reduce the incidence of systemic side effects [12,16,19,20,21].

A total of three clinical trials have been completed to date. The first Phase I/II study (IMMU-132-02) was conducted in patients with metastatic and refractory TNBC. The highly positive results and good tolerability at the 8–10 mg/kg dose led to accelerated approval of SG by the US Food and Drug Administration (FDA) in April 2020. With this approval, SG has the potential to become a third-line treatment for patients with unresectable metastatic TNBC who have received two or more prior systemic therapies [11,14,19,20].

The subsequent phase II/III clinical trial (ASCENT) was conducted with a similar sample as the IMMU-132-02 trial. They divided the participants into two groups and compared SG with standard chemotherapy, which was selected individually for each patient by the treating physician. After demonstrating a clear clinical benefit compared to standard chemotherapy (with a clinical benefit rate (CBR) of more than 45%), the FDA gave final approval in April 2021 for the routine use of SG in TNBC that is refractory to two or more prior systemic therapies [11,12,16,19,20,21].

The last completed Phase III trial (NeoSTAR) was completed in December 2023. This trial is the first to include a sample of patients with good prognosis TNBC treated with SG in the neoadjuvant setting, i.e., prior to radical or conservative surgery. The three clinical trials agreed that the most common adverse events of the drug, although manageable, were neutropenia, leukopenia, diarrhea, anemia, and febrile neutropenia [12,14,16,19,20]. In addition, a higher incidence of severe neutropenia was observed in patients with homozygous alterations in the liver enzyme uridine diphospho-glucuronyl transferase (UGT1A1) in the ASCENT trial [16,19].

The main objective of this systematic review of randomized clinical trials was to provide a comprehensive analysis of the efficacy and safety of SG as a monotherapeutic treatment in patients diagnosed with TNBC. In addition, a detailed comparison of the results obtained with SG with the standard treatment regimen of conventional chemotherapy currently used for this type of cancer was to be performed.

## 2. Materials and Methods

The literature and data analysis of this systematic review was conducted according to the guidelines of the PRISMA 2020 Working Group [28].

### 2.1. Research Question and Eligibility Criteria

The review was designed to answer the question, “Is sacituzumab govitecan an effective treatment in patients with TNBC”?

The papers considered for inclusion in this systematic review covered a period of the last 8 years, from January 2017 to March 2024. We primarily included clinical trials completed as of 20 March 2024, study arms, and analyses of each trial (IMMU-132-02, ASCENT, and NeoSTAR). In addition, articles or conference abstracts corresponding to cohorts or analyses subsequently performed as part of the clinical trial were selected.

During the initial title and abstract screening, articles written in languages other than English, Spanish, and German were excluded. Clinical trials that were not completed and were in the recruitment phase or did not have final statistical results as of 20 March 2024. In addition, other articles that compared SG with new immunotherapies or treatments other than standard chemotherapy were discarded. Articles that evaluated SG in the context of other drugs compared with chemotherapy were discarded because this systematic review evaluates SG in monotherapy. Also excluded were those that evaluated the results of the drug in cancers other than TNBC, even if they were Her2neu or hormone receptor-positive (HR+) CM. In the full-text article review phase, we excluded letters to the editor, personal opinions, books, book chapters, non-original reports, single cases, editorials, commentaries, or articles that did not contribute to or complement the objectives of the review.

### 2.2. Information Sources and Search Strategy

A search for relevant articles was conducted in Pubmed, Web of Science (WOS), and Cochrane databases between September 2022 and January 2024. The search strategy used Medical Subject Headings (MeSH) terms: “sacituzumab govitecan”, “breast cancer”, “triple negative breast cancer”, “guidelines”, “UGT1A1”, “clinical trial”, “HR”, and “adverse events”. These terms were combined using the Boolean operators “AND”, “OR”, and “NOT”. The search strategies are shown in Supplementary Table S1.

### 2.3. Study Selection

After the elimination of duplicate articles, an initial screening of titles and abstracts was performed, followed by a review of the full text. The initial screening was performed by two authors, M.C.-P. and M.P.-B., and the full text review was performed by all authors.

### 2.4. Data Extraction

For each article, all the information related to the drug was extracted, such as pharmacokinetics, pharmacodynamics, mechanism of action, molecular structure, main positive effects related to the results of TNBC, comparison of the same results with other standard therapies against TNBC, most common and serious side effects, and the impact that SG is causing as a new treatment pathway in the guidelines of this very aggressive type of cancer.

### 2.5. Synthesis Method

The synthesis process started by analyzing all the major findings and conclusions about the drug. Once the synthesis was completed, the findings and conclusions of each article were compared and will be discussed in the discussion of this systematic review.

## 3. Results

### 3.1. Articles Included in the Review

**Figure 2:**
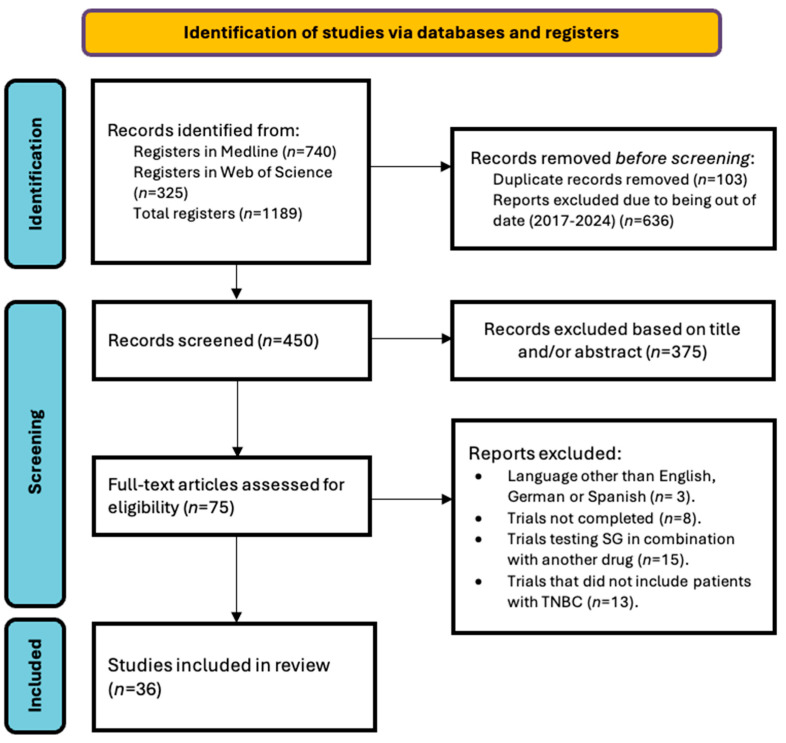
PRISMA flowchart of study selection process.

The initial search yielded a total of 1189 articles. A total of 36 articles were included in this review, each of which underwent an individual analysis based on the quality of the evidence and the information provided on the topic. Thus, all studies considered address the DCA sacituzumab govitecan and its efficacy as a third-line treatment in TNBC. Supplementary Table S2 shows the main characteristics of the 36 articles analyzed [26,27,29,30,31,32,33,34,35,36,37,38,39,40,41,42,43,44,45,46,47,48,49,50,51,52,53,54,55,56,57,58,59,60,61,62]. The flowchart in Figure 2 describes the screening and selection process.

This results section is organized first around the two most relevant clinical trials, detailing the data on drug efficacy in each trial, as well as the incidence and severity of adverse events and the conclusions drawn from them. Specific cohort analyses and subgroup study arms within these trials were included.

Other recent clinical trials that add to the available evidence on the efficacy and safety of sacituzumab govitecan in patients with good and poor prognosis TNBC and a German observational study are then reviewed. Finally, a section is dedicated to the review and recommendations for the management of adverse events in patients treated with this drug, highlighting relevant strategies and considerations for its future clinical application and even the development of a protocol.

### 3.2. IMMU-132 Clinical Trial

The IMMU-132 clinical trial (NCT01631552) is the first trial to test SG in humans. It includes phases I and II, so its main objective is to evaluate safety, determine the optimal and effective dose, identify potential side effects, and analyze the efficacy of the drug in a group of patients with various epithelial solid cancers, including a specific cohort of TNBC patients [27,29,30,31].

#### 3.2.1. Phase I (25 Patients—4 TNBC)→8, 10, 12, and 18 mg/kg

The study by Starodyub et al. [29] evaluated the efficacy and safety of SG in monotherapy as a potential treatment for patients with various epithelial solid tumors. The phase I IMMU-132 clinical trial enrolled 25 patients with advanced metastatic solid tumors refractory to at least two prior standard chemotherapy regimens indicated for each cancer type. Of these patients, four had TNBC, seven had pancreatic cancer, three had colorectal cancer, two had small cell lung cancer, and the remainder had other cancers [30,31]. However, patients with Gilbert’s disease (UGT1A1) were excluded, limiting the evidence on this aspect, as were patients with central nervous system metastases [29].

The sample was divided into four groups that received different doses of SG to determine effective and safe dosing. These doses were 8 mg/kg, 10 mg/kg, 12 mg/kg, and 18 mg/kg administered intravenously on days 1 and 8 of a 21-day cycle in 2–3 h infusions. In the subset of patients with TNBC, one patient received 10 mg/kg, two received 12 mg/kg, and one received 18 mg/kg SG [29,30,31,32].

Several controls were performed during the study, most notably Enzyme-Linked ImmunoSorbent Assay (ELISA) (anti-SN38 and antihRS7) to assess the potential development of autoimmunity against different fractions of the SG drug and IHC on archived tumor samples to analyze Trop 2 expression on tumor surfaces. The ELISA results showed the absence of development of autoimmunity against the drug after several cycles of SG treatment, suggesting a constant efficacy of the drug in pharmacological terms. On the other hand, IHC samples from 17 patients showed that Trop 2 was expressed in tumor cells in more than 75% of the sample. However, this sample was considered insufficient to establish a correlation between Trop 2 overexpression and the antitumor activity of SG [29].

Preinfusion or postinfusion prophylaxis with drugs such as acetaminophen, paracetamol, antihistamines, and dexamethasone was allowed. Antiemetic and antidiarrheal prophylaxis were initially prohibited to objectively evaluate these side effects, as they are known to occur in patients treated with irinotecan QT, although they will be considered in future phases of the clinical trial [29].

The results regarding SG posology showed different levels of related toxicity associated with the dose administered. At the 18 mg/kg dose, all patients experienced limiting toxicity with two cases of grade 4 neutropenia and one case of grade 2 diarrhea. At the 12 mg/kg dose, none of the patients experienced limiting toxicity at the first infusion, which initially led to this dose being considered the maximum tolerated dose. However, in repeated cycles, this dose proved toxic with the development of severe neutropenia. The doses of 8 and 10 mg/kg were found to be acceptable and effective not only in a first infusion but also in prolonged treatment, with no grade 4 toxicities and few cases of fatigue, neutropenia, diarrhea, and leukopenia, all maximum grade 3 [29]. Therefore, the maximum tolerated dose in a single infusion was determined to be 12 mg/kg, which is toxic in repeated cycles, while the best tolerated dose in the first cycle, allowing infusion of several repeated cycles with minimal toxicity, was 8 and 10 mg/kg, which would be further evaluated in subsequent phase I to III studies [29,30,31].

Adverse events (AEs) were observed, most commonly fatigue, nausea, alopecia, diarrhea, and grade 1–2 neutropenia, and were manageable with adequate prophylactic treatment [29,31]. In terms of efficacy outcomes, these were not the primary focus of this study, but two patients with TNBC who had also previously received CT with a topoisomerase I inhibitor to which they did not respond were observed to have a partial response (PR) in SG and a significant reduction in tumor size of 28% and 12%, respectively, in addition to a time to disease progression (TDP) of 8.5 months and 4.1 months, respectively [29].

#### 3.2.2. Phase I-II (178 Patients—53 TNBC)→8 and 10 mg/kg

After determining that the maximum tolerated dose in repeated cycles is 8 and 10 mg/kg [29], the following clinical trial was conducted by Ocean et al. [33] to evaluate the pharmacokinetics and safety of multiple cycles of SG as monotherapy at these intravenous doses in patients with metastatic epithelial solid cancers refractory to more than two prior standard therapies [30].

The sample consisted of 178 patients, 53 of whom had TNBC. These participants were assigned to two dose groups on a first-come, first-served basis: 81 received 8 mg/kg and 97 received 10 mg/kg. Patients with a diagnosis of Gilbert’s disease were excluded, but the incidence of the different UGT1A1 haplotypes in the entire sample was subsequently investigated [33].

In the control procedures during this study by ELISA technique, it was observed and confirmed that no anti-SG response was detected in the serum sample, indicating that resistance to the SG drug is not generated [30,33]. In addition, an IHC study was performed on 150 archived patient samples, showing high Trop-2 expression in 93% of samples and moderate expression in 82% of positive samples [33].

Prophylactic treatment was allowed as in the phase I trial, but this time primary prophylaxis with antiemetics and anticholinergics for diarrhea was allowed. In addition, hematopoietic growth factor was considered for the first time as secondary prophylaxis, i.e., after the first infusion, to treat neutropenia. Later, its use as primary prophylaxis will be allowed, but in phase I-II trials, this approach was chosen to objectively assess the incidence and severity of neutropenia [33].

The results of this second study showed that the safe dose with manageable and effective toxicity, providing objective responses and a good therapeutic index, is 10 mg/kg intravenously on days 1 and 8 in 21-day cycles [32,33]. SG-related AEs were observed in >90% of patients in both groups. However, although the 10 mg/kg group had a slightly higher incidence of grade >3 AEs such as diarrhea and neutropenia, there was no significant difference in grade 1–2 AEs between the two groups, which does not justify the use of 8 mg/kg doses as the starting dose. Even after the third dose of SG, the incidence of grade 3 neutropenia was 50% in the 8 mg/kg dose group and 42% in the 10 mg/kg dose group [33].

Regarding the pharmacokinetics of the drug, it has been shown that 50% of the payload of the IgG-bound SN-38 metabolite (>95% IgG-bound SN-38 and <5% free SN-38) is released daily, which, being bound, protects from glucuronidation and ensures sufficient persistence of SG in the organism [30]. The half-life of the cytotoxic is 11–14 h, and free Ab without SN-38 is metabolized in >100 h. The half-life of free SN-38 is not known, which theoretically corresponds to irinotecan chemotherapy [30,32,33].

Due to the hepatic metabolism of SG, treatment is not recommended in patients with moderate/severe hepatic insufficiency as determined by pathologic levels of bilirubin and transaminases (bilirubin ≤ 1.5 × upper limit of normal [ULN]; aspartate aminotransferase [AST] and alanine aminotransferase [ALT] ≤ 2.5 × ULN; and serum albumin ≥ 3 g/dL). However, no significant effects were found between drug pharmacokinetics and the following characteristics: mild/moderate/severe and even chronic renal insufficiency (RI) [30,32], age, sex, baseline albumin level, race, ECOG status, solid tumor type, UGT1A1 genotype, and Trop 2 expression level [32].

Regarding the genotypic study of UGT1A1, 146 patients were confirmed to have an alteration of this liver enzyme, of which 19 patients were homozygous type 28*28, 64 were heterozygous type 1*28, and 63 were wild type 1*1. Among these three groups, neutropenia was observed in 58%, 39%, and 38% of patients, and diarrhea in 16%, 8%, and 5% of patients, respectively [33]. Alterations in this liver enzyme were confirmed to influence the incidence and severity of AEs, but there were no significant differences between haplotypes and doses of 8 or 10 mg/kg, suggesting that dose did not influence the increased incidence of AEs in patients with different UGT1A1 genotypes [30,33]. However, genotyping of all patients to be treated with SG is not yet recommended, but in case of prediagnosis of the disease, these patients should be closely monitored [30,31].

Although the results regarding the efficacy of the drug are not the focus of this study, it can be concluded that the objective response rate (ORR) was 10% in the 8 mg/kg group and 22% in the 10 mg/kg group. In addition, the clinical benefit rate (CBR) was higher at the 10 mg/kg dose [33].

#### 3.2.3. Phase I-II (69 TNBC)

The following study by Bardia et al. [34] was a multicenter clinical trial to evaluate the efficacy and safety of SG in monotherapy. It focused primarily on ORR, with secondary consideration of progression-free survival (PFS) and overall survival (OS). In addition, we evaluated the incidence of AEs associated with the drug at the dose established in a previous study [33], which was 10 mg/kg intravenously on days 1 and 8 of 21-day cycles. Patients received an average of 14 doses per patient in 7 treatment cycles with an average follow-up of 16.6 months.

The sample consisted of 69 patients with advanced metastatic TNBC who were refractory to more than 1 prior standard treatment with CT. Patients with newly diagnosed and progressive brain metastases (BM) requiring corticosteroid therapy were excluded [34].

ELISA tests were performed to detect anti-SN38 or anti-hRS7 Ab in serum, and all results were negative, again confirming the absence of an immune response by the organism to the drug [33]. An IHC study was performed on archived samples from 48 patients, of which 42 (88%) showed moderate to strong Trop 2 staining, while 6 (22%) had weak or no staining. When these findings were compared with treatment efficacy, a trend toward prolonged PFS of 7.1 months was observed in patients with moderate to strong Trop 2 expression, as opposed to 3.1 months in those with weak or no expression [34]. Other studies have confirmed that higher Trop 2 expression correlates with better PFS and ORR rates, but with a shorter OS time. Nevertheless, the data obtained so far confirm that SG appears to be more effective than CT as a standard regimen [35]. Prophylactic treatment was approved with the same guidelines and drugs as in the previous study [33,34].

Efficacy results showed an overall 69.5% reduction in tumor burden in the 69 patients in the study. The ORR was 30% in a total of 21 patients, of which 19 had a PR and 2 had a complete response. Notably, 13 of the patients who objectively responded had a tumor size reduction in greater than 30% at the first imaging check 8 weeks after starting treatment, with a median time to objective response after the first infusion of 1.9 months. TB was 46% of the sample. The median PFS was 6 months (95% CI 5–7.3) and the median progression-free survival was 16.6 months (95% CI 11.1–20.6), of which 63 patients experienced progression and 33 died during these periods [34].

Forty-one percent of patients in the sample experienced grade 3 AEs, including neutropenia (39%), febrile neutropenia (7%), leukopenia (14%), anemia (14%), diarrhea (13%), vomiting (10%), and thrombocytopenia (3%). Three patients discontinued treatment after the sixth or seventh dose, and no deaths related to SG drug toxicities were reported [34].

#### 3.2.4. Phase I-II (408 Patients—108 TNBC)

In May 2018, both Wahby et al. [36] and Bardia et al. [37] submitted a proposal to conduct a fourth clinical trial with the goal of obtaining US FDA approval of the drug [36]. On 22 April 2020, the FDA granted accelerated approval for SG at a standard dose of 10 mg/kg based on the results of this clinical trial. This approval would allow SG to be used in a larger number of patients with a better prognosis and will facilitate the advancement of this drug into a Phase III trial [27,30,31,33,38].

The main objective of this clinical trial was to perform a comprehensive safety evaluation of SG in monotherapy [30,36]. In addition, the efficacy of the treatment was evaluated with a focus on ORR, response duration, TBC, PFS, and OR in patients with metastatic and advanced solid epithelial cancers [27,30,37,39].

The sample consisted of 408 patients with advanced solid epithelial cancer who had received a median of more than three prior standard therapies [30,36]. Ninety-eight percent of patients had received taxanes, and 86% had received anthracyclines. Within this sample, there is a cohort of 108 TNBC patients with a median age of 55 years [27,30,36,37].

The most relevant controls performed in this study included imaging tests for disease staging. Computed tomography and magnetic resonance imaging (MRI) scans were performed at baseline and then every eight weeks after initiation of SG treatment. If a favorable objective response was observed, further confirmation was required by another new imaging study performed at least four weeks after the initial finding [27,37].

Blood transfusions and hematopoietic growth factors were allowed after the first infusion as secondary prophylaxis for SG. Ninety-two percent of patients received primary prophylaxis, which included antidiarrheal atropine, antiemetics, anxiolytics, glucocorticoids, atropine, acetaminophen, and H1/H2 antihistamines [27,37].

In the analysis of the cohort of 108 TNBC patients by Bardia et al. [37], the most common Grade 1–2 AEs occurring in more than 25% of the sample were diarrhea, fatigue, nausea, neutropenia, anemia, alopecia, constipation, rash, decreased appetite, and abdominal pain. In addition, a greater than 5% incidence of neutropenia, anemia, and grade 3 diarrhea was observed. Severe AEs were observed in 35 patients in the sample and included febrile neutropenia (7%), vomiting (6%), nausea (4%), diarrhea (3%) and dyspnea (3%). However, no grade 3 peripheral neuropathy was observed. Neutropenia was a very common and serious AE, leading to discontinuation in 48 patients (44%), and dose reduction in 33%. There were four deaths during treatment in this sample, but none were attributed to SG but rather to disease progression, as the deaths occurred more than 30 days after the last SG infusion [27,30,36].

The median objective duration of response (DOR) in the 36 responding patients was 7.7 months [36,37,39]. Of the total responding patients, 55.6% had a DOR of more than 6 months but less than 12 months, while 16.7% had a DOR of more than 12 months. The median PFS was 5.5 months (95% CI 4.1–6.3) and the median OS was 13 months (95% CI 11.2–13.7). In addition, the median response time was 2 months (95% CI 1.6–13.5) [36,37]. The results were analyzed in different subgroups according to age, metastatic disease, and number of prior therapies, among other factors, and it was concluded that these aspects did not lead to significant differences in response rates [27,30,37].

UGT1A1 haplotypes were analyzed by Sakar Wahby et al. [36] in the complete sample of 343 patients and showed grade 4 neutropenia in different haplotypes: 26% incidence in homozygotes, 13% in heterozygotes, and 11% in the wild type. These results support the FDA recommendation that patients who are known to carry an altered UGT1A1 haplotype should be followed closely in order to detect AEs early and avoid the development of severe forms of neutropenia, diarrhea, anemia, and major toxicities in general [27,30,31]. However, despite the guidelines provided by the FDA, it is not recommended to systematically perform UGT1A1 haplotyping in patients initiating treatment with SG, despite the fact that this genetic alteration predisposes the patient to severe AEs. In addition, the appropriate dosage for homozygous 28*28 patients is currently unknown, and an individualized approach is recommended [31,39].

#### 3.2.5. Phase I-II (495 Patients—144 TNBC)

The latest phase I-II clinical trial of the IMMU-132 group by Bardia et al. [38] included 408 patients in their sample and also expanded the inclusion of participants for a comprehensive analysis of the incidence and severity of SG-associated AEs, which was their main objective. The rates corresponding to the drug efficacy analysis were evaluated, and similar results were found in the cohort of 144 patients with TNBC compared to the previous study [36,37], prior to the inclusion of more patients.

The expanded sample included a total of 495 patients with metastatic epithelial solid tumors refractory to more than two prior standard chemotherapy regimens, of whom 144 had TNBC. Of the patients, 98.6% had stage IV disease, 67% were female, 82% were Caucasian, and the median age of the group was 61 years [30,38].

The controls used in this study were identical to those used in the previous section, as they were part of the same clinical trial but included the expansion of patients [36,37,38].

Hematopoietic growth factor was used in 175 patients (35.4%), while blood transfusions were required as secondary prophylaxis in 46 patients (9.3%), always after the first SG infusion. In addition, 424 patients (85.7%) received primary prophylaxis, with antiemetics used in 62.4% of the sample, corticosteroids in 54.7%, antihistamines in 29.3%, and antacids in 26.7% [38].

The results presented regarding AEs show that 483 patients (97.3%) discontinued treatment at some point, mainly due to disease progression (335 patients) or AEs (41 patients). In addition, there was one death due to pneumonia secondary to bronchial aspiration, which was not related to SG [38]. The most common grade 1–2 AEs were nausea (62.6%), neutropenia (57.8%), diarrhea (56.2%), fatigue (48.3%), alopecia (40.4%), and febrile neutropenia (5.5%). On the other hand, grade 3 or higher AEs, although less frequent, included nausea (3.6%), diarrhea (7.9%), grade 3 (28.9%) and grade 4 (13.5%) neutropenia, anemia (10.3%), and grade 3 (4.2%) and grade 4 (1%) febrile neutropenia. No grade 3 or higher peripheral neuropathy or ocular toxicity was observed in association with SG [30,38].

The results with respect to age, number of prior therapies, and metastatic disease, among others, and again concluded that there was no significant relationship between these subgroups and SG efficacy or tolerability. In summary, patients with different types of solid epithelial cancers should respond similarly regardless of their age or prior treatment history [27,30,36,37,38].

Drug efficacy remained consistent in the TNBC cohort with an ORR of 33.3% and a DOR of 7.7 months. All other staging and outcomes were unchanged compared to the pre-study extension article [27,30,36,37,38].

All patients were tested for the presence of UGT1A1 haplotypes. Of the 403 patients with any genetic variation, 36% were 1*1 wild-type, another 36% were heterozygous and 9% homozygous, while the remainder had other variants. The incidence of neuropenia was 33.3% in 1*1 patient, 38.8% in 1*28 patients, and 60.9% in 28*28 patients. The incidence of diarrhea was 54.8% in 1*1 patient, 51.7% in 1*28 patients, and 60.9% in 28*28 patients. The incidence of anemia was 37.3% in 1*1 patient, 31.7% in 1*28 patients, and 50% in 28*28 patients [30,38].

Regarding treatment discontinuation, 98.3% of 1*1, 96.7% of 1*28, and 97.8% of 28*28 patients discontinued treatment at some point, although no temporality is described. Therefore, it is concluded that homozygous 28*28 patients were more likely to experience AEs of anemia and neutropenia. However, the incidence of diarrhea was similar in all three haplotypes. Regardless of the incidence of AEs in the UGT1A1 subtypes, most patients had to discontinue the drug at some point, with no difference between haplotypes [30,37,38].

### 3.3. ASCENT Clinical Trial

The multicenter, open-label, randomized phase III ASCENT clinical trial by Bardia et al. [40] was designed to compare two study groups: one assigned to treatment with SG as monotherapy and the other with standard CT at the choice of each patient’s treating physician [30,39,41,42,43]. The primary objective was to evaluate the efficacy (ORR, PFS, OS…) and safety of SG compared to single-agent CT [30,39,40,42]. This was performed to obtain full FDA approval to grant the drug for use in clinical practice, in addition to completing their investigations in different cohorts [40,43].

A total of 529 patients with advanced and recurrent metastatic TNBC were enrolled, all of whom had received at least two prior standard therapies (eribulin, vinorelbine, capecitabine, or gemcitabine), including taxanes in the overall sample (taxanes 100%, anthracyclines 82%, carboplatin 66%, PD1/PDL1 27%, PARP inhibitors 7%, paclitaxel, and cyclophosphamide) [26,30,40,41,42,44]. However, for a more precise analysis of drug PFS, a sample of 468 patients with TNBC and without BM was selected [30]. This is because the presence of BM is associated with higher disease severity in terms of prognosis, which could bias the SG results. Therefore, only data from patients without BM were included in the statistical analysis of efficacy and AEs. However, one of the cohorts studied subsequently included only patients with BM [30,40].

The final sample consisted of 468 patients randomized 1:1 to two treatment groups. One group, consisting of 235 patients, would receive SG at a standard dose of 10 mg/kg on days 1 and 8 in 21-day cycles. The other group, consisting of 233 patients, would receive single-agent CT. The choice of chemotherapeutic agent was made on an individual basis by the treating physician (eribulin 54% (126 patients), vinorelbine 20% (47 patients), capecitabine 13% (31 patients), or gemcitabine 12% (29 patients). It was established that there would be no switch of treatment group in case of disease progression to avoid any bias in SG assessment [26,30,39,40,41,42,43,44,45].

Patient follow-up included imaging (CT/MRI) every 6 weeks for the first 36 weeks and then every 9 weeks until disease progression or end of treatment. Although patients were not systematically genotyped for UGT1A1 haplotypes, those with a pre-treatment diagnosis were closely monitored. In addition, strict caution was exercised in prescribing UGT1A1 inducers or inhibitors to all patients, regardless of whether their haplotype mutation carrier status was known or not [40,41,44,45].

Inducers included drugs such as carbamazepine, phenytoin, cortisol, phenobarbital, spironolactone, streptozotocin, rifampicin, St. John’s wort, ritonavir, efavirenz, lamotrigine, primidone, and zidovudine. On the other hand, inhibitors included amitriptyline, ketoconazole, ketoprofen, atazanavir, propofol, nilotinib, pazopanib, valproate, and olaparib, among others [30,39].

The prophylaxis used in this study was very similar to that used in the IMMU-132 trial. It included hematopoietic growth factors after the first SG infusion, which were administered in 49% of the SG-treated patients and 23% of the CT-treated patients. In addition, antiemetics, anticholinergics, glucocorticoids, antihistamines, and other previously mentioned drugs were used [26,30,39,40,44,45].

The efficacy of GS in 468 patients with TNBC without BM was manifest in several aspects. The ORR for SG was 35%, with a PR rate of 31% and a CR of 4%, compared to 5% observed in the CT-treated group, where PR was 4% and CR was 1% [26,30,31,40,43,44,45]. CBR was 45% for SG and 9% for CT [40]. The median PFS for the SG group was 5.6 months (95% CI 4.3–6.3), while for the CT group it was 1.7 months (95% CI 1.5–2.6) (hazard ratio for disease progression or death, 0.41; 95% CI, 0.32 to 0.52; *p* < 0.001). For OS, the median SG was 12.1 months (95% CI 10.7–14), while the median CT was 6.7 months (95% CI 5.8–7.7) (hazard ratio for death, 0.48; 95% CI, 0.38 to 0.59; *p* < 0.001) [26,30,39,40,41,42,43,44,45]. The results showed that the benefit of SG over CT in PFS remained consistent across all pre-specified subgroups, including patients older than 65 years, patients with more than three prior standard therapies, including PD1/PDL1 inhibitors, patients with an initial diagnosis of TNBC, patients with metastatic disease, and other cohorts listed below. In addition, the DOR in the SG group was 6.3 months compared to 3.6 months in the CT group [40]. At 24 months, the long-term OS rate was 22.4% in the SG arm compared to 5.2% in the CT arm [39].

The most common Grade 1–2 AEs were neutropenia (SG: 63%; CT: 43%), diarrhea (SG: 59%; CT: 12%), nausea (SG: 57%; CT: 26%), alopecia (SG: 46%; CT: 16%), fatigue (SG: 45%; CT: 30%), and anemia (SG: 34%; CT: 24%). The most common grade 3 or higher AEs were neutropenia (SG: 51%; CT: 33%), leukopenia (SG: 10%; CT: 5%), diarrhea (SG: 10%; CT: <1%), anemia (SG: 8%; CT: 5%), and febrile neutropenia (SG: 6%; CT: 2%). Notably, no grade 3 neuropathy was observed [26,30,39,40,41,45].

AEs were also reported in the safety population, which included all patients with or without BM who received more than one dose of SG or CT. This cohort included a total of 482 patients (SG: 258 patients; CT: 224 patients). The most common AEs were neutropenia (SG: 52%; CT: 33%), diarrhea (SG: 11%; CT: 0.4%), anemia (SG: 8%; CT: 5%), and febrile neutropenia (SG: 6%; CT: 2%) [43].

A higher incidence of both mild and severe AEs was observed in the SG group compared to the CT group, although they were considered manageable with prophylactic therapy [30,39,40]. A quarter of patients in both groups required dose reductions due to AEs [30]. In addition, only 2 to 5% of patients in each group experienced drug discontinuation, with no significant differences between the groups. Of note, at the time of data cutoff, 15 of 235 patients were continuing SG due to lack of disease progression, whereas in the CT group, no patients were continuing treatment as all patients without exception had disease progression. There were three deaths related to AEs in each group, but in the SG group they were not related to the drug but to the underlying TNBC progression itself. The causes were two cases of respiratory failure and one case of obstructive pneumonia, the latter occurring 14 days after the last drug infusion. In contrast, in the CT arm with eribulin, one death was related to the drug as it was attributed to neutropenic sepsis [30,40,41,43,45].

#### 3.3.1. TNBC Patients with BM Cohort

This study cohort by Dieras et al. [46] analyzed the SG effect in patients with metastatic TNBC and BM refractory to a median of five prior standard chemotherapeutic regimens (*n* = 61), representing 12% of the 529 patients enrolled in the ASCENT clinical trial [30,40]. They were randomized into two groups: 32 patients received SG and 29 patients received CT at the physician’s discretion (*n* = 32 vs. *n* = 29). The treatment guidelines, long-term follow-up, and prophylactic measures in the clinical trial were the same for all patients, both with and without BM. However, strict eligibility criteria were applied to patients with BM, including stable brain MRI for at least four weeks prior to randomization and stable disease for at least two weeks without active anticonvulsant treatment. In addition, safe discontinuation or dose reduction corticosteroids prior to randomization was required [46].

The ORR was 3% in the SG group and 0% in the CT group. PFS was 9.4% in the SG arm and 3.4% in the CT arm, with stable disease in 47% and 31% of patients, respectively. Median PFS was 2.8 months (95% CI 1.5–3.9) in the SG group and 1.6 months (95% CI 1.3–2.9) in the CT group. The median OS was 6.8 months (95% CI 4.7–14.1) in the SG group and 7.5 months (95% CI 4.7–11.1) in the CT group. The DOR was 6.7 months in the SG group, while it was not calculable in the CT group [30,46].

The most common AEs of any grade were fatigue (SG: 63%; CT: 52%), diarrhea (SG: 50%; CT: 13%), neutropenia (SG: 43%; CT: 35%), nausea (SG: 43%; CT: 26%), decreased appetite (SG: 30%; CT: 17%), leukopenia (SG: 33%; CT: 22%), anemia (SG: 23%; CT: 35%), alopecia (SG: 23%; CT: 13%), and constipation (SG: 23%; CT: 22%). There were no SG-related deaths [40,46].

In conclusion, the results suggest that SG showed greater benefit than CT in terms of ORR and PFS, although it showed inferior OS. Importantly, despite the difference in median OS between the two treatments, the maximum interquartile range of SG is 3 months greater than that of CT [46].

#### 3.3.2. Cohort of Patients Aged > 65 Years (<65 Years and >75 Years)

The following cohort study by Kalinsky et al. [47] included patients older than 65 years with metastatic TNBC without BM and refractory to more than 2 prior standard therapies (*n* = 90). Efficacy was compared between SG (*n* = 44) and CT (*n* = 46), noting that the SG group had 50% ORR, 61% CBR, median PFS of 7.1 months, and median OS of 15.3 months versus 0% ORR, 9% CBR, median PFS of 2.4 months, and median OS of 8.2 months in CT.

In patients younger than 65 years (*n* = 378), SG also outperformed CT in terms of ORR (31% vs. 6%), CBR (41% vs. 9%), median PFS (4.6 months vs. 1.7 months), and median OS (11.2 months vs. 6.6 months) [40,47]. Patients older than 75 years (*n* = 7) received only SG, of which 2 patients had a PR and 4 patients had stable disease for more than 6 months and one for less than 6 months. No CRs were observed in this patient cohort [47].

In the cohort of patients older than 65 years, both the SG and CT groups experienced AEs of all grades in similar proportions. However, some AEs led to dose reductions in some patients, 35% in the SG group and 33% in the CT group. The most common causes of these reductions were neutropenia (SG: 14%; CT: 25%), fatigue (SG: 10%; CT: 4%), diarrhea (SG: 6%; CT: 0%), and nausea (SG: 4%; CT: 0%). Importantly, AEs were less common in the cohort of patients younger than 65 years, with 19% in the SG group and 24% in the CT group. In addition, serious AEs leading to discontinuation were reported in patients older than 65 years (SG: 2%; CT: 2%) and more frequently in patients younger than 65 years (SG: 5%; CT: 6%). No AE-related deaths were reported in patients older than 65 years in any treatment group [40,47].

#### 3.3.3. Cohort of Patients with UGT1A1 Deficiency

The toxic metabolite of SG, SN-38, is the same as that of the chemotherapeutic agent irinotecan and is mainly metabolized through the hepatobiliary pathway via the enzyme UGT1A1. Genetic variants that affect the function of this enzyme may prolong the presence of the toxic metabolite, thereby exacerbating AEs [45]. Rugo et al. [45] analyzed 250 patients with altered UGT1A1 variants, which were part of the total sample of 468 patients studied by Bardia et al. [40].

The homozygous 28*28 haplotype (*n* = 34) showed dose reduction due to AEs in 35% of cases, with a mean time to dose reduction of 1.8 months. The heterozygous 1*28 haplotype (*n* = 96) also showed dose reductions in 19% of cases, with a mean time to reduction of 2.7 months. On the other hand, the wild-type 1*1 haplotype (*n* = 113), which unlike the previous ones has normal enzymatic activity, showed dose reduction in 18% of cases, with a similar time to reduction as 1*28 [39,45].

The most common AEs were grade >3 neutropenia (59% in 28*28; 47% in 1*28; 57% in 1*1), febrile neutropenia (18% in 28*28; 5% in 1*28; 3% in 1*1), anemia (15% in 28*28; 6% in 1*28; 4% in 1*1), and diarrhea (15% in 28*28; 9% in 1*28; 10% in 1*1). These data suggest that the homozygous 28*28 haplotype had a higher incidence of these AEs compared to the other haplotypes. However, the incidence of other AEs such as nausea, vomiting, constipation, fatigue, alopecia, and decreased appetite was similar among all haplotypes [39,45].

These findings highlight an apparent higher incidence of grade >3 AEs in patients with UGT1A1 variants compared to those without; however, the FDA does not recommend a systematic UGT1A1 screening protocol [45].

#### 3.3.4. Cohort of Patients with Strong/Moderate/Low Trop 2 Expression

The following cohort of the ASCENT clinical trial analyzed by Bardia et al. [48] was designed to analyze Trop 2 Ag expression in TNBC tumors and evaluate its association with clinical outcomes. Biopsy or surgical tissue samples of the primary tumor were collected to determine Trop 2 expression by IHC studies, which was categorized into four levels: strong (+3), moderate (+2), weak (+1), and null (+0).

Of the 468 patients with metastatic TNBC without BM, Trop 2 expression was positive in 290 cases. Sixty-four percent of the SG-treated group (*n* = 151) showed Trop 2 positivity, with strong (+3) expression in 56%, moderate (+2) in 26%, weak (+1) in 18%, and null (0+) in 7 patients [39,48].

Efficacy results varied according to Trop 2 expression. The ORR in SG-treated patients was 44%, 38%, and 22% in strong, moderate, and weak expression, respectively. Median PFS in the SG-treated arm was 6.9 months, 5.6 months, and 2.7 months for strong, moderate, and weak expression, respectively. Similarly, the median PFS in the SG arm was 14.2 months, 14.9 months, and 9.3 months for strong, moderate, and weak expression of Trop 2 on the tumor surface, respectively.

#### 3.3.5. BRCA1/2 Patient Cohort

BRCA1/2 mutation screening was performed at the beginning of the study by Bardia et al. [40]. These results allowed them to evaluate the effects and outcomes of SG in monotherapy in patients with a positive mutation [48]. The resulting cohort consisted of 292 mutation-positive patients, equally divided between the SG drug treatment (*n* = 149) and standard single-agent CT (*n* = 143) groups. The objective of evaluating these patients was to determine if there was an association between the mutation and efficacy as reflected in clinical outcomes.

BRCA1/2 mutation-positive patients treated with SG had a median PFS of 4.6 months compared to 2.5 months for those treated with CT. Patients treated with SG had a median PFS of 15.6 months, while those treated with CT had a median PFS of 4.4 months. The ORR was 19% in the SG group and 6% in the CT group [48].

However, results for patients without BRCA1/2 mutations showed that those treated with SG had a median PFS of 4.9 months compared to 1.6 months for those treated with CT. The SG-treated patients had a median PFS of 10.9 months, while the CT-treated patients had a median PFS of 7 months. The ORR was 33% in the SG group and 6% in the CT group [48]. In both groups, there were numerically superior results with SG compared to CT for both patients with and without germline BRCA1/2 mutations. Therefore, they conclude that there is no significant difference between the effect of SG and the presence or absence of this mutation [48].

#### 3.3.6. Cohort of Patients Without an Initial Diagnosis of TNBC

In this sub-analysis by O’Shaughnessy et al. [49] of the ASCENT clinical trial, the impact of SG was examined in two groups of patients: those with an initial diagnosis of TNBC and those whose tumors developed TNBC after initially being hormone receptor or Her2neu positive. This transformation limits standard treatment options such as hormonal therapy or trastuzumab and forces the use of chemotherapy in TNBC.

The objective of this cohort is to evaluate the effects of SG in patients with and without initial diagnosis of TNBC in terms of ORR, PFS, OS, and adverse events. The background of this study arm is to understand how different BC subtypes respond to SG, as late-diagnosed TNBC tumors may still have some estrogen, progesterone, or Her2neu membrane receptor, as opposed to a patient diagnosed with TNBC from the onset of disease [49].

This phenomenon of altered hormone and Her2neu receptors in BC recurrences and metastases, the pathophysiology of which remains a mystery but may influence the efficacy of SG, is increasingly being discussed. A retrospective analysis of 993 tissue samples from primary and recurrent BC tumors [49] showed that a significant proportion of these tumor tissue samples showed changes in receptor status, with a higher frequency of positive to negative changes. These alterations are associated with less favorable clinical outcomes and imply worse survival because they experience a higher mutability rate compared to patients who maintain their receptor-positive status. Therefore, the aim is to show whether these patients have a greater or lesser advantage than those who have always had receptor-negative disease.

The sample analyzed consisted of 146 patients with TNBC and without BM who did not have TNBC at initial diagnosis, out of a total of 468 patients [49]. With a median age of 55 years and 63% with an ECOG index of 1, indicating a good physical condition with respect to the disease [49]. These patients were divided into two groups: 70 received SG and 76 received CT [44,49].

In terms of outcomes, patients without an initial diagnosis of TNBC treated with SG demonstrated an ORR of 31%, with a CR rate of 1% and a PR rate of 30%. The median PFS was 4.6 months, with 13% of patients maintaining stable disease after 12 months of treatment. Median OS was 12.4 months, with 57% and 27% of patients alive at 12 and 18 months, respectively [49].

In contrast, patients without an initial diagnosis of TNBC treated with CT showed an ORR of 4%, with a CR rate of 1% and a PR rate of 3%. The median PFS was 2.3 months, with 3% of patients maintaining stable disease at 12 months. The median OS is 6.7 months, with 18% and 8% of patients surviving at 12 and 18 months, respectively [49].

On the other hand, patients with an initial diagnosis of TNBC treated with SG showed an ORR of 36%, with a CR rate of 5% and a PR rate of 31%. The median PFS was 5.7 months, with 20% of patients maintaining stable disease after 12 months of treatment. Median OS was 12.1 months, with 50% and 32% of patients alive at 12 and 18 months, respectively [49].

O’Shaughnessy et al. [49] found that SG was more beneficial than CT in patients without an initial diagnosis of TNBC. However, no significant differences in SG drug efficacy were observed between patients with or without an initial diagnosis of TNBC, suggesting that the timing of TNBC diagnosis did not influence treatment response. Severe AEs were similar in both groups, although a small improvement was observed in patients without an initial diagnosis of TNBC. No deaths related to SG AEs were reported [49].

#### 3.3.7. Cohort of Patients Who Relapsed <12 Months After Their Last Standard Therapy

Carey et al. [50] analyzed this next cohort, which aimed to evaluate the impact of SG in patients with metastatic TNBC without BM who experienced recurrence in less than 12 months after receiving standard neoadjuvant CT in the metastatic setting and prior to their enrollment in the ASCENT clinical trial. The basic premise of this analysis is that patients who relapse in such a short period of time represent a group of patients whose disease is considered more aggressive, having failed to respond satisfactorily to prior long-term chemotherapy. The objective was to compare the impact of SG with CT in this specific subset of patients. The sample was divided into two treatment groups, one with SG and the other with CT, with a total of 33 and 32 patients, respectively. The same controls and posology were used as in the original study [50].

Patients treated with SG had an ORR of 30% compared to 3% in patients treated with CT. In addition, the ORR was 42% and 6% in the SG and CT groups, respectively. The median PFS was significantly higher in the SG group (5.7 months vs. 1.5 months) compared to the CT group, as was the median PFS (10.9 months vs. 4.9 months). The DOR was 6.7 months in the SG arm, while it was not calculable in the CT arm. Regarding AEs, the safety profile of SG was consistent with previous reports, with no drug-related deaths [50]. SG results were superior to those of CT in this specific cohort, with efficacy rates similar to those observed in the overall ASCENT trial sample [50].

#### 3.3.8. Black Patient Cohort

TNBC disproportionately affects black women and is characterized by higher incidence in younger women, higher recurrence rates, higher tumor aggressiveness, and a lower response to standard therapies compared to other ethnicities [5,7,8]. To address this scenario, Carey et al. [51] analyzed the impact of SG in this cohort of 62 African–American patients with metastatic TNBC refractory to more than two prior standard treatments. They were divided into two equal groups: one received SG (*n* = 28) and the other received CT (*n* = 34).

In terms of drug efficacy, an ORR of 32% was observed in the SG group and 6% in the CT group, with a CBC of 43% and 15%, respectively. Median PFS was 5.4 months for SG and 2.2 months for CT, while median PFS was 13.8 months for SG and 8.5 months for CT. Treatment DOR was 5.4 months for SG and 2.2 months for CT [51].

The most common grade 3 or higher AEs were neutropenia (SG: 48%; CT: 42%), anemia (SG: 12%; CT: 6%), leukopenia (SG: 8%; CT: 16%), and febrile neutropenia (SG: 8%; CT: 3%). No patients in the SG arm discontinued treatment due to AEs, while 3% of patients in the CT arm discontinued treatment due to AEs. There were no SG-related deaths in the cohort of African American patients [51]. Although the proportion of African–American patients in the ASCENT trial was limited (12%), those treated with SG had a clinical benefit comparable to the overall results of the entire sample, along with a manageable safety profile, such that SG was considered equally effective regardless of patient race [51].

#### 3.3.9. Health-Related Quality of Life Analysis

Several publications [52,53,54,55] reported a health-related quality of life (HRQoL) analysis of TNBC patients treated with SG versus CT from the ASCENT clinical trial. The EORTC QLQ-C30 questionnaire was administered at baseline, before each treatment cycle, and 4 weeks after study discontinuation due to progression, AEs, or other reasons. This questionnaire covers various health-related aspects such as global health status, physical, emotional, cognitive, and social functioning, as well as the presence of symptoms such as fatigue, nausea, vomiting, pain, dyspnea, insomnia, loss of appetite, constipation, and diarrhea, and finally the presence of financial difficulties.

A total of 419 patients with metastatic TNBC without BM, with a median age of 54 years [52,53,54], completed the questionnaire at baseline and at least once thereafter to perform the benefit analysis [55]. Of these, 236 received SG and 35% showed an objective clinical response (CR/PR), while 183 received CT and only 6% showed an objective clinical response [52,53,54].

Baseline questionnaire scores showed a similar response in both groups [53,54], but over the course of the study, SG-treated patients showed a marked improvement in several aspects of HRQoL compared to CT-treated patients. Specifically, improvements in global health status, physical and emotional functioning, and reduced symptomatic impact of fatigue, pain, dyspnea, and insomnia were observed in the SG-treated patients. However, SG-treated patients experienced more negative symptom impact from diarrhea, nausea, and vomiting compared to CT-treated patients, and these findings persisted throughout the study. Despite the higher incidence of diarrhea in the SG group, this did not translate into a negative impact on patients’ overall perception of health or general functioning [52,53,54,55]. On the other hand, the SG group was shown to have greater financial difficulties than the CT-treated group [54]. In addition, the mean time to the first sign of objective clinical deterioration was longer in patients treated with SG than in those treated with CT [55].

Finally, both SG responders and non-responders showed significant improvements in HRQoL compared to those treated with CT. Overall, SG was associated with greater improvement and delayed worsening of HRQoL scores compared to CT, supporting its clinical benefit and favorable development as a treatment for TNBC [43,52,53,54,55].

#### 3.3.10. Analysis of Patients Who Progressed After Treatment with SG

At the conclusion of the ASCENT trial, Cortés et al. [56] conducted a study to evaluate the response to subsequent treatment of TNBC patients who had previously been treated with SG during the trial but whose disease progressed. Their post-progression treatment was evaluated as well as its effect on OS.

Of the 267 patients who received SG in the clinical trial, 222 patients (83%) had to discontinue treatment due to disease progression. Upon progression, patients were offered the choice of CT-based post-progression therapy or no treatment. Those who accepted therapy received it after a median of 5.4 months. The post-CT group included 163 patients who received various treatments, including eribulin (*n* = 70), carboplatin (*n* = 34), capecitabine (*n* = 34), atezolizumab (*n* = 15), and others. On the other hand, 59 patients decided not to receive any post-progression therapy [56].

The group that received post-progression therapy had a median OS from randomization of 13.4 months (eribulin 14.1 months; carboplatin 13.6 months; capecitabine 16.5 months; atezolizumab 14.9 months), whereas those who did not receive such therapy had a median OS of 7.3 months [56].

They also evaluated median PFS from the end of SG treatment for disease progression, which was 7.9 months in the group that received post-progression therapy (eribulin 8.4 months; carboplatin 8.9 months; capecitabine 8.6 months; atezolizumab 8.9 months) and 2 months in those who received no treatment. These values indicate that patients started to progress 4–5 months after the start of SG in the ASCENT trial [57].

### 3.4. Other Clinical Trials

In May 2023, Xu et al. [58] published the analysis of a phase IIb clinical trial conducted in China evaluating the efficacy and safety of SG in 80 patients with metastatic TNBC refractory to more than two prior standard therapies and without evidence of BM. SG was administered intravenously at a dose of 10 mg/kg in 21-day cycles until disease progression or unacceptable toxicity. A median of 16 doses and 8 cycles were administered for a median SG of 5.62 months, while the median CT was 2.33 months. It was observed that 37 patients discontinued treatment due to disease progression, 18 died, and 47 discontinued for other reasons. Lymph node and lung metastases were the most common.

An ORR of 38.8% (PR 36.3%; CR 2.5%; stable disease 43.8%) and a CBR of 82.5% were observed. Tumor shrinkage was observed in 61 patients, including >30% shrinkage in 40 patients, with a median response time of 1.51 months and a DOR of 5.59 months. Median PFS was 5.55 months, and median OS at 3, 6, and 9 months were 93.8%, 82.5%, and 68%, respectively. These data include the more severe subgroups with lung and liver metastases [58].

Regarding AEs, the entire sample experienced them, with the most common being neutropenia (85%), anemia (82.5%), leukopenia (81.3%), vomiting (55%), and nausea (50%). Grade 3 or higher AEs were neutropenia (62.5%), leukopenia (48.8%), and anemia (21.3%). One death was reported during the third treatment cycle due to possible hypovolemic shock related to SG AEs with a history of severe diarrhea and myelosuppression. No prophylactic measures were described in the study [58].

In July 2023, Reinisch et al. [59] published the results of an observational study conducted at the German medical center Kliniken Essen-Mitte. This was the first European study after EMA approval to use SG in routine clinical practice. The study analyzed the response of 43 patients with de novo or metastatic TNBC treated with SG as second- or third-line therapy.

Of the patients, 8 were diagnosed with de novo metastatic TNBC, 22 had no initial diagnosis of TNBC, and 23 had this diagnosis previously. All patients received standard treatment with taxanes, platinum, gemcitabine, eribulin, and PD1/PLD1 inhibitors [59]. Regarding posology, a dose of 10 mg/kg SG was administered according to FDA and EMA guidelines. However, the dose was reduced in 13 patients due to toxicities, and a dose of 7.5 mg/kg was initiated in 8 patients based on clinical judgment and comorbidities that could increase the severity of AEs [59].

The most common Grade 1–2 AEs were alopecia (90.7%), neutropenia (32.6%), diarrhea (37.2%), fatigue (34.9%), anemia (34.9%), elevated transaminases (30.2%), dyspnea (16.3%), and vomiting (7%). The most common grade 3 AEs were neutropenia (27.9%), diarrhea (18.6%), febrile neutropenia (4.7%), anemia (4.7%), nausea (4.7%), elevated transaminases (4.7%), fatigue (2.3%), vomiting (2.3%), and dyspnea (2.3%) [59].

Primary prophylaxis with G-CSF was used in 11 patients at high risk of developing myelosuppression due to a history of febrile neutropenia secondary to CT. The same drug was used as secondary prophylaxis in four patients who developed grade 3 neutropenia or febrile neutropenia during treatment with G-CSF. Diarrhea was treated with loperamide as secondary prophylaxis. Cold caps were offered to prevent alopecia, but no clinical benefit was observed in the five patients who used them [59]. In terms of efficacy, the median PFS was 5 months and the median OS was 13.1 months. There were eight deaths due to disease progression unrelated to SG AEs [59].

In December 2023, Spring et al. [60] published the results of the NeoSTAR phase III clinical trial, which evaluated the use of SG as neoadjuvant therapy in 50 patients with localized TNBC and favorable prognosis. These patients were stage I (*n* = 13), II (*n* = 26), and III (*n* = 11) lymph node negative, and none had stage IV disease. Only nine patients were BRCA mutation positive.

Patients received SG in the neoadjuvant setting at a dose of 10 mg/kg on days 1 and 8 of 21-day cycles, with a minimum requirement of 4 cycles, which were completed by 49 patients. After receiving the 4 cycles, 29 patients underwent surgery to prevent recurrence. Of this group, 13 patients received adjuvant CT due to residual disease, while 1 patient did not receive treatment due to metastatic recurrence. Another 20 patients received additional neoadjuvant chemotherapy prior to surgery, of whom 7 had a CR and did not require adjuvant CT, but 13 did. One patient did not complete all four cycles due to early progression [60].

The overall ORR was 64%, with specific ORRs of 54%, 69%, and 64% in stage I, II, and III patients, respectively. Of the 9 BRCA mutation-positive patients, 5 had CR or PR and 4 had stable disease. Among patients without a BRCA mutation, 27 had CR/PR, 12 had stable disease, and only 1 had disease progression. The pCR rate was 50%, 27%, and 18% in stage I, II, and III patients, respectively. As for 2-year PFS, it was 95% in the overall sample, with 100% survival in pCR-positive patients and 92% in patients with residual disease, with 2 deaths in the latter group [60].

The most common AEs were nausea (82%), fatigue (76%), alopecia (76%), rash (48%), and neutropenia (44%). Genotyping of UGT1A1 haplotypes was not performed, but evaluation of Trop 2 expression as a tumor biomarker was included. Both strong/moderate (*n* = 28) and low (*n* = 17) expressions were found to have no significant impact on predicting pathologic complete response [60].

### 3.5. Adverse Event Management and Recommendations

Following the FDA approval of SG in April 2020, several recommendations have been made regarding the management of more common and severe AEs [27,31,39].

Nausea and vomiting are very common after SG administration [27,30,36,37,39], with a median time to onset of 8 days after infusion [45]. Because SG is considered a highly emetogenic agent [39], premedication with 5-HT3 antagonists (ondansetron; palonosetron) and dexamethasone is recommended as primary prophylaxis [31,39,45]. In high-risk patients, the addition of NK1 antagonists (aprepitant; fosaprepitant) and olanzapine to standard prophylaxis may be considered. It is also advisable to offer outpatient prophylaxis with drugs such as ondansetron, prochlorperazine, or olanzapine and to educate patients about the warning signs of this AE [31,39]. SG should be discontinued in the event of grade 3 or greater nausea or vomiting, with the possibility of resuming treatment once symptoms have resolved to grade 1 [39].

Diarrhea and cholinergic syndrome affect approximately 60% of patients treated with SG, compared to 12% of patients treated with CT in Phase III trials. These AEs typically manifest approximately 19 days after SG infusion and may last approximately 5 days [45]. Preventive prophylactic treatment is not recommended, but intervention is recommended if patients experience acute diarrhea or cholinergic syndrome during or within the first 24 h after treatment infusion [31,39]. For these events (severe abdominal cramps, severe diarrhea, post-infusion sweating, and excessive salivation), intravenous atropine is usually administered during the episode [31,39,45] and during future SG infusions [39]. In the case of delayed diarrhea, i.e., more than 24 h after the drug infusion, an infectious cause should be ruled out and oral loperamide should be started along with fluid and electrolyte replacement to prevent dehydration [31,39,45]. If symptoms are not controlled, it is recommended that the dose of SG be reduced by 25%, 50%, or discontinued after the first, second, and third events, respectively. If SG is discontinued due to grade 3 or greater diarrhea, it may be resumed if symptoms resolve to grade 1 in less than 3 weeks [39].

Neutropenia, a common AE secondary to treatment with SG, affects 63% of patients in any grade, with 51% in grade 3 or higher. The onset of neutropenia is approximately 21 days after SG infusion, with a median duration of 6 days. This condition led to dose delays in 46% of patients and dose reductions in 11% [39,45]. Dose reductions were used in cases of severe neutropenia, fever, or other grade 3 or 4 AEs. For delays or reductions, a protocol of 25% dose reduction at the next infusion after the first event, 50% at the second event, and discontinuation at the third event, regardless of prophylaxis, was used [31]. Although primary prophylaxis with granulocyte colony-stimulating factor (G-CSF) was not recommended in early clinical trials, it was used as secondary prophylaxis in patients who subsequently developed neutropenia [31,39]. There was no established protocol for its use [39]. Indications for secondary prophylaxis include grade 4 neutropenia lasting more than 7 days, fever greater than 38.5 °C, grade 3 or 4 neutropenia that delays dosing for 2–3 weeks to recover to grade 1, or any grade from day 1 or day 8 after infusion [31]. The approximate treatment is to prescribe filgrastim for 3–4 days after day 1, never administered within 24 h before or after infusion, followed by pegfilgrastim from day 8 [39,45].

Clark et al. from the Mayo Clinic [61] conducted a retrospective analysis of the management of neutropenia in TNBC patients treated with SG, evaluating the use of G-CSF. They studied 67 patients who received more than one dose of SG and experienced neutropenia, infusion delays, and treatment with G-CSF. Sixty-three percent received G-CSF during treatment, more frequently in the first two cycles. Primary prophylaxis was given in 12 patients with no history of neutropenia, but the reason was not specified. Secondary prophylaxis was given in 81% of patients with SG and G-CSF, with 26 patients having treatment delays due to neutropenia. The median number of SG cycles was 5 in all patients and in those with neutropenia who received G-CSF, with a range of 1 to 25, while those who did not receive prophylaxis had a median of 4 cycles, with a range of 1 to 19. The lack of an established protocol highlights the need for future trials of individualized prophylactic treatment.

Febrile neutropenia, with an incidence of 6%, is considered an oncologic emergency. Low-risk patients initially receive broad-spectrum intravenous antibiotics, with consideration of oral therapy if stabilized in approximately 4 h. In high-risk patients, IV broad-spectrum antibiotics are given urgently, followed by hospitalization. Once stabilized, SG can be continued with dose adjustments and support with G-CSF. Importantly, febrile neutropenia does not warrant study discontinuation, allowing patients to resume SG cycles once this adverse effect has been treated [39].

Anemia affected approximately 50% of treated patients, and it is important to first rule out other causes such as bleeding or colorectal tumors secondary to TNBC [39]. Patients who were symptomatic or had a hemoglobin level of less than 7 g/dL received packed red blood cell transfusions as secondary prophylaxis [31,39]. In cases of severe or persistent anemia, dose reduction or discontinuation was considered [39].

In the IMMU-132 clinical trial, hypersensitivity reactions were observed in 37% of cases, manifesting within the first 24 h after SG infusion. Therefore, it is recommended that patients be premedicated with antipyretics such as acetaminophen and H1/H2 antihistamines. In patients with a history of allergic reactions, the addition of corticosteroids to primary prophylaxis prior to each infusion should be considered. In addition, it is important that patients are educated about warning signs such as swelling of the face, tongue, lips, appearance of urticaria, or difficulty breathing, among others, and report these symptoms to the center immediately [31].

Alopecia is a common AE in patients receiving SG, although its specific incidence and severity have not been determined. Nevertheless, the use of scalp cooling cap devices is permitted during treatment infusion, although their efficacy is not known [31]. In patients treated with standard CT agents such as anthracyclines and taxanes, these devices have been shown to reduce the rate of alopecia by 50% around the hair follicle by decreasing the absorption of the chemical at that level [39]. However, the efficacy of these devices in patients undergoing SG treatment is still under investigation [31,39].

If epidermal eruptions develop, topical corticosteroids and oral H1 antihistamines may be considered. However, in cases of uncertainty or complications, referral to the dermatology service is preferred [31].

## 4. Discussion

### 4.1. Relationship Between Trop 2 Expression, SG Efficacy and the Bystander Effect

The hypothesis of the “bystander effect” associated with SG represents a potentially promising mechanism to improve therapeutic efficacy in patients with solid tumors that overexpress Trop 2. According to this theory, the toxic metabolite of the drug, SN-38, has the property of being permeable to the cell membrane, which could induce apoptosis of specific tumor cells once SG has been internalized into a target cell. Subsequently, this toxic metabolite could diffuse to neighboring tumor cells to induce their apoptosis, whether they express Trop 2 or not [22,23,24].

This feature suggests that SG could be effective in any type of epithelial solid tumor expressing Trop 2, regardless of the level of overexpression of this biomarker. That is, even in tumors with weak overexpression (+1) of Trop 2, SG could have a significant therapeutic impact because the cytotoxicant could diffuse across tumor membranes without the need for specific binding to the Trop 2 receptor. This implies that SG could act as a localized CT, affecting both tumor cells with strong (+3) Trop 2 expression and those with weak (+1) expression [22,23,24]. This hypothesis is based on theoretical considerations about the drug, and until it is evaluated in clinical trials, its validity in TNBC will not be confirmed.

Starodyub et al. [29] provide IHC studies in 17 patients with advanced solid epithelial cancers and show that although Trop 2 expression was positive in more than half of the samples analyzed, the specific distribution of expression of this biomarker in the two TNBC patients in this sample was not detailed in the study. However, the efficacy results in these two patients showed a significant benefit with an objective reduction in tumor size.

The lack of detailed information on Trop 2 expression in TNBC patients in this study [29] limits our ability to draw definitive conclusions about the relationship between biomarker expression and response to SG. Nevertheless, the positive results observed in these patients suggest that SG could be effective regardless of the level of Trop 2 expression, as suggested by the bystander theory. This does not exclude the possibility that SG could be equally effective in tumors with low Trop 2 expression, which would broaden its potential clinical application.

It is important to note that this analysis is based on a small sample size (*n* = 2), and a detailed analysis of the correlation between Trop 2 expression and treatment response in TNBC patients was not performed. Thus, according to this study, the conclusion regarding this question raised needs further studies and phases with larger samples [29].

The analysis by Ocean et al. [33] provides valuable information on Trop 2 expression and safe and effective dosing of SG in patients with metastatic solid epithelial cancers. The IHC study performed on archived samples showed a high prevalence of positive Trop 2 expression, and in addition, most of these samples showed moderate to strong (+2, +3) expression of the biomarker. Although the study did not provide specific details on the number of samples corresponding to patients with TNBC, it is inferred that at least some of the patients with this disease could have positive and elevated Trop 2 expression, given the high percentage of positivity observed in the overall sample [33].

The primary objective of this phase of the study was to determine the safe and effective dose of SG to achieve objective tumor response; therefore, the efficacy of SG in patients with TNBC was not specifically evaluated. Therefore, no specific response rate data were provided for this subset of patients [33].

Although the findings on the specific expression of Trop 2 in TNBC patients and the evaluation of the efficacy of SG treatment in these patients are limited, the results of IHC analysis suggest that this biomarker is overexpressed in several types of epithelial solid cancers, including TNBC [33]. Therefore, it should be analyzed in further studies to evaluate its efficacy according to strong, moderate, or weak expression.

The results of the analysis by Bardia et al. [34] consistently support the high expression of Trop 2 in this type of cancer, suggesting its relevance as a possible therapeutic target and even as a predictive marker. IHC analysis of the samples revealed that most of them showed moderate to strong Trop 2 staining, while a very low percentage showed weak or no staining. These findings raise the possibility of considering Trop 2 expression as a factor to be considered in the design of new DCAs and as a potential indicator of treatment response. In other words, there may be a correlation between high Trop 2 expression and the therapeutic benefit provided by SG.

However, the interpretation of these results is complicated by the bystander effect, which is not fully understood. Although it is postulated that even a small amount of Trop 2 in tumor cells could induce apoptosis and affect neighboring cells, the extent to which the amount of Trop 2 influences the efficacy of SG treatment remains to be determined. This raises questions about the clinical utility of assessing Trop 2 expression as a predictive marker [34,35].

On the other hand, the discussion of the clinical relevance of the amount of Trop 2 in tumor cells should be approached with caution. Although in this sample we observed a significant benefit in median PFS in patients with high and low expression of the biomarker, as well as a favorable ORR in those with higher Trop 2 expression, several additional considerations are important to keep in mind. Median OS was lower in patients with higher Trop 2 expression compared to those with lower expression. This raises questions about the efficacy of SG in patients with different levels of Trop 2 expression and suggests that other biological and clinical factors may influence outcomes. It is also worth noting that the sample of patients with low Trop 2 expression is limited, making it difficult to draw definitive conclusions about its impact on treatment efficacy. Furthermore, it is proposed that SG may act differently in patients with different levels of Trop 2 expression and may vary according to other biological and clinical factors that have not been fully explored [34,35].

Although the high expression of Trop 2 in TNBC suggests its potential as a therapeutic target and predictive marker, the relationship between the level of Trop 2 and response to SG treatment is complex and requires further evaluation in future studies with larger samples and a thorough analysis of other factors that may influence treatment efficacy. However, recent evidence suggests that TNBC patients do indeed have elevated Trop 2 expression in their normal state and that this leads to improved efficacy of SG [34,35].

Bardia et al. [48] evaluated Trop 2 expression in TNBC patients without BM treated with SG. The results showed that the majority of these patients had increased Trop 2 expression, with 56%, 26%, and 18% of them showing strong (+3), moderate (+2), and weak (+1) expression, respectively. These findings again confirm that TNBC, as a solid epithelial cancer, has a high overall Trop 2 expression [39].

The efficacy results of SG in terms of ORR, PFS, and OS showed that indeed, the higher the Trop 2 expression, the better the clinical outcomes. A significant difference was observed between the strong/moderate expression groups and the weak expression group [39,48]. These findings challenge the “bystander effect” theory and suggest that Trop 2 expression is indeed an important factor influencing the clinical outcomes and prognosis of TNBC patients treated with SG.

The question that arises is whether IHC testing to assess Trop 2 expression prior to offering SG in routine clinical practice would be warranted. Although the FDA has not provided data or recommendations in this regard, it is important to note that SG continues to demonstrate very favorable outcomes in TNBC patients, regardless of their Trop 2 expression, compared to the less favorable outcomes of standard CT [39,48].

Therefore, although assessment of Trop 2 expression could provide additional information regarding potential response to SG, the overall positive results of the drug in TNBC patients support its use regardless of Trop 2 expression. Consequently, testing for Trop 2 expression as part of routine clinical practice may not be warranted as SG remains a superior therapeutic option to standard CT in this patient population [39,48].

### 4.2. Pharmacokinetics and Study of Autoimmunity Against SG by ELISA Controls

Throughout the various phases of the IMMU-132 clinical trial, extensive ELISA controls were performed to evaluate the potential occurrence of autoimmunity against SG components. Specifically, the presence of Ab against the cytotoxic metabolite SN-38 (Ab Anti SN-38) and against Ab itself binding to the Trop 2 receptor (Ab Anti hRS 7) was evaluated. The results obtained consistently showed the absence of development of autoimmunity against the drug, even after multiple cycles and doses of SG. This finding, documented in several studies [29,33,36,37,38], suggests that SG does not induce an abnormal immune response in the body, supporting its safety and tolerability in patients with TNBC and other epithelial solid cancers. This suggests that SG is well tolerated by the human body and does not induce a significant autoimmune response. This aspect is crucial because it suggests that SG can be safely administered repeatedly as needed for the treatment of patients with TNBC and other solid epithelial cancers. Thus, it is hypothesized that if SG ceases to be effective, it is more likely to be due to tumor progression rather than an adverse immune system response to the drug components [29,30,32,33,36,37,38].

The half-life of the cytotoxic SN-38 bound to the IgG Ab of the drug is 11–14 h, due to the protection provided by the Ab against hepatic metabolism by glucuronidation. On the other hand, the Ab without the cytotoxic is metabolized in approximately 100 h. Although the exact half-life of free SN-38 is not known, it is known that in this form it corresponds to the topoisomerase I inhibitor irinotecan. However, since the cytotoxic metabolite is not bound to an Ab, as in the case of SG, its metabolism is much faster, and it remains in the body for a shorter time. This difference in pharmacokinetics between free SN38 and SN38 bound to SG supports the therapeutic efficacy of SG as it remains in the body for a longer period of time, which may increase its potential to affect the tumor more effectively. This fact correlates with the results of the phase I study by Starodyub et al. [29], where the two TNBC patients treated with SG who experienced PR and tumor shrinkage had previously received standard topoisomerase I inhibitor treatments and still progressed on these therapies [30,32,33,36,37,38].

The discussion of the pharmacokinetics of SG is important because it highlights the fact that the antibody is cleared in more than 100 h, but this does not appear to affect its therapeutic efficacy or induce drug resistance. This provides reassurance regarding the repeated administration of SG, as it is not expected to induce resistance or tolerance in patients. Furthermore, comparison with CT irinotecan suggests that SG may be more effective because it remains in the body for a longer period of time, increasing its potential to affect the tumor more effectively [30,32,33,36,37,38].

### 4.3. Alteration of Liver Enzyme UGT1A1 and Its Effect on SG-Treated Patients

Alterations in the liver enzyme UGT1A1, which is responsible for the glucuronidation and metabolism of the drug SG, raise important clinical considerations regarding its impact on the development, incidence, and severity of treatment-related AEs. In Allyson J. Ocean’s phase I-II clinical trial [33], patients with a prior diagnosis of Gilbert’s disease, an inherited disorder that results in impaired hepatic metabolism due to a deficiency of the enzyme UGT1A1, leading to the accumulation of bilirubin and other components, as in the present case, were excluded. However, at the end of the study, all patients were genotyped, and a high incidence of patients with a change in the UGT1A1 enzyme was found in the sample that was subsequently included [30,31].

It is puzzling that despite the initial exclusion of patients with Gilbert disease, a high incidence of alterations in the UGT1A1 enzyme was found in the final study sample. This discrepancy raises questions about the effectiveness of the initial exclusion and suggests that the prevalence of alterations in this enzyme may be higher than expected in the population with TNBC and solid epithelial cancers. Therefore, it appears that Gilbert’s disease is only one manifestation of a more generalized alteration in UGT1A1 enzyme function in these patients [30,31,33].

On the other hand, results from the IMMU-132 and ASCENT clinical trials suggest that alterations in the UGT1A1 enzyme may have a significant impact on the incidence and severity of AEs associated with SG. A high incidence of AEs such as neutropenia, diarrhea, and anemia was observed in patients with altered UGT1A1 haplotypes, especially in those with the homozygous haplotype (28*28) with a difference of 10–20%. However, all haplotypes had to discontinue SG treatment due to evidence of AEs in more than 95%. The temporality of this event is not described, so we cannot objectify whether the homozygous haplotype would discontinue treatment earlier than the heterozygous or wild type, as it would be logical to interpret. In the study cohort of Rugo et al. [45] of the ASCENT clinical trial, it was shown that these patients also underwent dose reductions, with the homozygotes in a higher proportion and in a much shorter mean time to reduction than the other two haplotypes. We can therefore assume that the homozygotes also discontinued treatment earlier, although ultimately the other haplotypes would do the same. These findings suggest that the identification of patients with alterations in this enzyme is crucial for planning close monitoring during SG treatment and prevention of AEs [27,30,31,36,38,39].

On the other hand, Sathe et al. [32] conducted a study focusing on comorbidities that could affect drug pharmacokinetics. They found that, among other things, the UGT1A1 genotype had no significant effect on drug pharmacokinetics, efficacy, or AEs, which was contradicted by the results of subsequent clinical trials.

Despite the clinical importance of these observations, the FDA does not recommend prior systematic screening for UGT1A1 alterations in patients receiving SG. However, the European Society for Medical Oncology recommends genotyping for this alteration in patients with metastatic colorectal cancer who will initiate CT with irinotecan, another agent that is also metabolized by the UGT1A1 pathway. This discrepancy raises the question of whether systematic screening for UGT1A1 should be recommended in all patients initiating treatment with cytotoxic agents such as SG, especially given its high potential to cause serious AEs [30,31,38,39,45].

The high prevalence of UGT1A1 alterations in TNBC patients and their impact on the incidence and severity of SG-associated AEs underscores the importance of identifying these patients prior to treatment. Although current guidelines may be inadequate in this regard, the results of clinical trials support the need to consider systematic UGT1A1 screening in clinical practice to ensure adequate monitoring and prevention of AEs [30,31,38,39,45].

### 4.4. Different Efficacy Results in Different Clinical Trial Cohorts

In the IMMU-132 clinical trial, the lack of a standard of care control group to compare outcomes with SG treatment is a major limitation in evaluating drug efficacy. Although the observed results were favorable, the lack of a comparator makes it difficult to accurately interpret the relative benefits of SG compared to standard therapies in TNBC. Nevertheless, when results were analyzed in different subgroups according to age, metastatic disease, and number of prior therapies, no significant differences in response rates were observed [27,30,36,37].

In the phase III ASCENT trial, Bardia et al. [40] addressed this issue by directly comparing two groups of TNBC patients. This time, patients with brain metastases were excluded due to their worse prognosis to avoid bias in the drug results. The results were encouraging, especially in the cohort of patients without brain metastases, where SG showed a significant benefit compared to standard CT [26,30,39,41,43,45].

Although differences in survival and other parameters have been observed between cohorts, such as those with brain metastases, those older than 65 years, or those with BRCA1/2 mutations, one constant has prevailed: SG consistently showed a clear clinical improvement compared to standard therapies. These results suggest that SG may represent a valuable therapeutic option for a wide variety of TNBC patients, regardless of their demographic profile or genetic characteristics, which are specified in each cohort study in this review [26,30,40,44,47,49,50,51].

It should be noted that while these results are promising, they come from patient populations with a generally poor prognosis, advanced stages of disease, and limited survival expectations. However, the efficacy observed in these populations suggests an even greater potential for SG when evaluated in cohorts of patients with a more favorable prognosis [30,38]. Therefore, the results obtained in these early-phase clinical trials support further investigation of SG in patient samples with a more optimistic prognosis, as demonstrated in the NeoSTAR clinical trial. These findings suggest a promising role for SG in the treatment of TNBC and justify the need for future clinical trials to evaluate its efficacy in a wider variety of clinical settings and patient populations [30,38,59,60].

### 4.5. Quality of Life in Relation to Patient Health and Its Association with the Incidence of AEs

The discrepancy between the incidence of AEs and perceived HRQoL in patients treated with SG versus CT in the ASCENT clinical trial raises an interesting discussion. Despite observing a higher incidence and severity of AEs in the SG-treated group, HRQoL questionnaires show a significant improvement in several aspects, including global health status, physical functionality, and emotional state [26,30,39,40,41,45,52,53,54,55].

This finding may be due to several factors. It is speculated that stricter prophylaxis in the SG group may have contributed to this improvement, although the rapidity of clinical deterioration in the CT group suggests that if prophylaxis had been available, it would have been equally early and preventive. However, these are only speculations, and there are no concrete data on the prophylaxis administered in the CT-treated group, so we do not have sufficient data to support this theory [33].

Prophylaxis against neutropenia prior to the first infusion was not allowed in the ASCENT clinical trial by Bardia et al. [40] in order to better assess the effect of the drug on this AE. However, in the observational study by Reinisch et al. [59], primary prophylaxis with G-CSF was allowed in high-risk patients, resulting in a significantly lower incidence of neutropenia compared to previous clinical trials where prevention of this AE was not allowed. All of this suggests that it is critical to protocol the management of neutropenia, both with primary prophylaxis and with preemptive initial dose reductions. Although possible management strategies are suggested in treatment guidelines, there is still no established protocol, underscoring the need for further research to develop clear and effective approaches to mitigate AEs associated with these treatments [31,39,45].

The discrepancy between the incidence of AEs and perceived quality of life in patients treated with SG versus CT in the ASCENT clinical trial raises important questions about the influence of prophylaxis, the rate of clinical deterioration, and the need for specific protocols for managing AEs. These findings underscore the complexity of balancing therapeutic efficacy and tolerability in the treatment of metastatic triple-negative breast cancer [26,30,31,39,40,41,45,52,53,54,55].

### 4.6. Methodological Flaws in Clinical Trials

Olivier and Prasad [62] performed a critical analysis of the ASCENT clinical trial. This highlighted potential biases and methodological errors that may have influenced the results in favor of SG over standard CT.

One of the main biases identified is the lack of blinding due to the open-label design of the trial, which allowed both patients and investigators to know which treatment they were receiving. This lack of blinding may exaggerate the effect of the experimental group. In addition, the withdrawal of consent from patients who were to receive CT due to a lack of blinding shows how this aspect may have influenced the allocation of patients to treatment groups [62]. Lack of blinding also occurred in the phase I-II IMMU-132 clinical trial, where the two SG treatment groups at doses of 8 and 10 mg/kg were assigned on a first-come, first-served basis [33].

The early discontinuation of the clinical trial is also of concern. The recommendation to stop the trial due to early evidence of efficacy may have inflated the magnitude of clinical benefit and possibly overestimated PFS, although it is important to consider that SG is a more stable and relevant endpoint in this setting [62].

Similarly, shortcomings have been noted in the control group that received standard CT, where not all patients received standard recommended treatments in TNBC, such as platinum and anthracyclines. This may have underestimated the results of the control group and biased the results towards SG [26,30,40,41,42,44,62]. Furthermore, the improvement observed in patients treated with eribulin, a non-standard treatment for TNBC, suggests the need to further investigate its efficacy compared to other treatments [62].

Finally, inconsistencies in AE management recommendations and protocols are highlighted, such as the lack of dose reduction, albeit with prophylactic G-CSF treatment, in the SG group after the first episode of febrile neutropenia, in contrast to the dose reduction applied in the CT group without prophylactic treatment after the first episode. These differences in the management of AEs may have influenced the observed survival rates and the incidence of AEs. In addition, the only treatment-related death occurred in the CT group, raising questions about the effectiveness of AE management strategies in both groups [27,31,39,62].

A critical analysis of the ASCENT clinical trial highlights the importance of considering potential biases and methodological errors when interpreting study results. These findings underscore the need for future research to confirm the efficacy and safety of SG compared to standard CT in the treatment of metastatic TNBC [62].

## 5. Conclusions

The FDA has granted accelerated approval for SG at a standard dose of 10 mg/kg. Studies reviewed indicate that this dose, administered intravenously on days 1 and 8 of 21-day cycles, is safe and effective.

Differential expression of Trop 2 correlates with prolonged progression-free survival (PFS), supporting SG efficacy. Variants in the liver enzyme UGT1A1 influence the incidence and severity of adverse events (AEs), especially in the homozygous haplotype (28*28). Close monitoring of patients with this genetic alteration is recommended to prevent AEs, although systematic screening is not recommended.

SG results show significant clinical benefit with improvements in ORR, PFS, OS, and CBR and a significant reduction in tumor size compared to standard chemotherapy. Patients treated with SG experienced improved health-related quality of life (HRQoL) compared to those treated with CT.

The benefit of SG did not differ significantly by age (>65 years and <65 years), metastatic disease, number of prior therapies, race, initial diagnosis of triple-negative breast cancer (TNBC), presence of brain metastases, recurrence less than 12 months after last standard therapy, Trop 2 status, BRCA mutation-positive, and other cohorts. In all cases, SG showed a consistent clinical benefit over CT.

SG has a higher incidence of AEs than CT; of particular concern is the higher incidence of neutropenia and diarrhea, which sometimes led to discontinuation of treatment with SG. The use of hematopoietic growth factors and other prophylactic measures was common, although there is no standardized management protocol for AEs associated with SG.

In conclusion, the results support the efficacy and safety of SG as a treatment for triple-negative breast cancer at a standard dose of 10 mg/kg. However, further studies are needed to establish management protocols for AEs and to optimize their clinical use.

## Data Availability

No new data were created or analyzed in this study.

## Supplementary Materials

The following supporting information can be downloaded at: https://www.mdpi.com/article/10.3390/cancers16213622/s1, Table S1: Search strategies; Table S2: Main characteristics of each study analyzed.
