# Supplementary material for: Sacituzumab Govitecan in Triple Negative Breast Cancer: A Systematic Review of Clinical Trials
Source: Cancers (Basel). 2024 Oct 27;16(21):3622. doi: 10.3390/cancers16213622 (PMC11545346; doi:10.3390/cancers16213622)
Supplement: Supplementary file 1 [file cancers-16-03622-s001.zip › cancers-3231207-supplementary.pdf]

Sacituzumab Govitecan In Triple Negative Breast Cancer. A Systematic Review

Supplementary material

Table S1. Search strategies

**Medline**	Sacituzumab govitecan/, Breast cancer/, Triple negative breast cancer/, Guidelines/, UGT1A1/, Adverse events/. (Sacituzumab govitecan*) AND (Sacituzumab govitecan* AND Breast cancer*) AND (Sacituzumab govitecan* AND Breast cancer* AND Triple negative breast cancer*) AND (Sacituzumab govitecan* AND Breast cancer* AND Guidelines*) AND (Triple negative breast cancer* AND UGT1A1* AND Sacituzumab govitecan*) AND (Sacituzumab govitecan* AND Adverse events* AND Breast cancer*)
**Web of Science**	Sacituzumab govitecan/, Triple negative breast cancer/, Clinical trial/. (Sacituzumab govitecan* AND Clinical trial*) AND (Sacituzumab govitecan* AND Triple negative breast cancer*) AND (Triple negative breast cancer* AND Guidelines*)
**Cochrane**	Sacituzumab govitecan/, Triple negative breast cancer/, HR/. (Sacituzumab govitecan*) AND (Sacituzumab govitecan* AND Triple negative breast cancer* NOT HR*)

Table S2: Main characteristics of each study analyzed.

<table>
<tr><th colspan="5">PUBLICATION DETAILS</th></tr>
<tr><th>REFERENCE</th><th>AUTHORS</th><th>DATE</th><th>TYPE OF STUDY</th><th>CLINICAL TRIAL</th></tr>
<tr><td>[26]</td><td>Bardia A, et al.</td><td>23.10.2023</td><td>Systematic Review</td><td>IMMU-132 and ASCENT</td></tr>
<tr><th colspan="5">RESEARCH CONTENT</th></tr>
<tr><th>OBJECTIVES</th><th>SAMPLE</th><th colspan="2">RESULTS</th><th>CONCLUSIONS</th></tr>
<tr>
<td>Analysis of efficacy, incidence and severity of various AEs and HRQoL presented by the SG drug.</td>
<td>(n = 529)
Patients with recurrent advanced metastatic TNBC and with/without BM. Previously treated with more than two prior standard therapies.

TNBC without BM (n = 468):
- SG group (n = 235).
- CT group (n = 233)</td>
<td colspan="2">IMMU-132:

ORR of SG: 33%.
Median PFS of OS: 5.5 months.
This study led to the approval of SG by the FDA in the U.S. and the initiation of the Phase III ASCENT trial to evaluate the drug as a second- and third-line treatment for TNBC.

ASCENT:

Efficacy:

ORR: SG (35%); CT (5%)
Median PFS: SG (5.6 m); CT (1.7 m)
Median OS: SG (12.1 m); CT (6.7 m)

AEs:

<table>
<tr><th></th><th>SG</th><th>CT</th></tr>
<tr><td>Neutropenia</td><td>51%</td><td>33%</td></tr>
<tr><td>Diarrhea</td><td>10.5%</td><td><1%</td></tr>
<tr><td>Anemia</td><td>8%</td><td>5%</td></tr>
<tr><td>Febrile neutropenia</td><td>6%</td><td>2%</td></tr>
</table>
- No grade >3 neuropathy or interstitial lung disease, and no deaths related to SG treatment were reported.</td>
<td>ASCENT is the first Phase III study of an ADC to show a significant improvement in PFS and OS over CT in pretreated TNBC, confirming the clinical activity and safety profile of SG monotherapy.</td>
</tr>
</table>

<table>
<tr><th colspan="5">PUBLICATION DETAILS</th></tr>
<tr><th>REFERENCE</th><th>AUTHORS</th><th>DATE</th><th>TYPE OF STUDY</th><th>CLINICAL TRIAL</th></tr>
<tr><td>[27]</td><td>Goldenberg DM, et al</td><td>12.05.2020</td><td>Systematic Review</td><td>IMMU-132</td></tr>
<tr><th colspan="5">RESEARCH CONTENT</th></tr>
<tr><th>OBJECTIVES</th><th>SAMPLE</th><th colspan="2">RESULTS</th><th>CONCLUSIONS</th></tr>
<tr><td>Collect and compare all information relevant to the IMMU-132 trial and each of its phases.</td><td>(n = 108)

Patients with metastatic TNBC who have failed more than three standard therapies.</td><td colspan="2">The IMMU-132 trial was approved by the FDA based on published results from the original study of 108 patients with TNBC.
Efficacy:
• ORR: 33.3% (IC 95% 24.6 - 43.1).
• PR: 33 patients
• CR: 3 patients
• CBR: 45.4%.
• Median DOR: 7.7 months (95% CI 4.9 - 10.8)
• Median PFS: 5.5 months (95% CI 4.1 - 6.3)
• Median PFS: 13.0 months (95% CI 11.2 - 13.7)
Grade 3 adverse events
• Nausea: 26%.
• Diarrhoea: 8%.
• Neutropenia: 42% // Febrile neutropenia: 9.3%.
• Treatment discontinuation due to AEs: 44%.
• Treatment discontinuation due to AEs: 2.8%.
• Prophylactic treatment: 92%.</td><td>The results regarding the efficacy of the SG drug are very encouraging and surpass the known results of CT in these patients as the standard of care to date. However, a phase III clinical trial comparing the two treatments is needed.</td></tr>
</table>

<table>
<tr><th colspan="5">PUBLICATION DETAILS</th></tr>
<tr><th>REFERENCE</th><th>AUTHORS</th><th>DATE</th><th>TYPE OF STUDY</th><th>CLINICAL TRIAL</th></tr>
<tr><td>[29]</td><td>Starodub AN, et al.</td><td>05.05.2015</td><td>Phase I clinical trial</td><td>IMMU-132</td></tr>
<tr><th colspan="5">RESEARCH CONTENT</th></tr>
<tr><th>OBJECTIVES</th><th>SAMPLE</th><th colspan="2">RESULTS</th><th>CONCLUSIONS</th></tr>
<tr>
<td>Determine the maximum tolerated and optimal doses and the tolerability of SG as monotherapy at the following doses:
- 8 mg/kg
- 10 mg/kg
- 12 mg/kg
- 18 mg/kg</td>
<td>(n = 25)

Patients with advanced metastatic solid tumours that have failed more than two prior standard therapies, as indicated by cancer type.
TNBC (n = 4):

- 10 mg/kg (n = 1)
- 12 mg/kg (n = 2)
- 18 mg/kg (n = 1)</td>
<td colspan="2">18 mg/kg: All patients developed limiting toxicity after the first infusion of SG.
12 mg/kg: No limiting toxicity was observed after the first infusion, but this is a toxic dose in repeated cycles as patients experienced grade 3-4 neutropenia at this dose.
10-8 mg/kg: There were no limiting toxicities after the first infusion or in repeated cycles, so this is considered the maximum tolerated dose for multiple cycles of treatment. There were no grade 4 AEs.
Efficacy:
<table>
<tr><th></th><th>1 TNBC patient</th><th>1 TNBC patient</th></tr>
<tr><td>Tumor size reduction</td><td>28%</td><td>12%</td></tr>
<tr><td>TDP</td><td>8.5 months</td><td>4.1 months</td></tr>
</table>
• Stable disease: 16 patients
AEs:
• 8-10 mg/kg grade 3: Fatigue (n = 3), neutropenia (n = 2), diarrhea (n = 1) y leukopenia (n = 1)</td>
<td>12 mg/kg is the maximum tolerated dose in a single infusion.
8 and 10 mg/kg are the maximum tolerated doses in repeated cycles and have been selected for further Phase II studies to determine the final safe and effective dose.
Two patients with TNBC who were previously treated with topoisomerase I inhibitors to which they did not respond had positive responses to treatment with SG.</td>
</tr>
</table>

<table>
<tr><th colspan="5">PUBLICATION DETAILS</th></tr>
<tr><th>REFERENCE</th><th>AUTHORS</th><th>DATE</th><th>TYPE OF STUDY</th><th>CLINICAL TRIAL</th></tr>
<tr><td>[30]</td><td>Schreiber AR, et al</td><td>11.10.2021</td><td>Systematic Review</td><td>IMMU-132 and ASCENT</td></tr>
<tr><th colspan="5">RESEARCH CONTENT</th></tr>
<tr><th>OBJECTIVES</th><th>SAMPLE</th><th colspan="2">RESULTS</th><th>CONCLUSIONS</th></tr>
<tr><td>Collect and compare all information relevant to the IMMU-132 (Phase I and I/II) and ASCENT studies.</td><td>IMMU-132:
- Phase I (n = 25)
- Phase I/II (n = 69)
- Phase I/II (n = 408 and 108 with TNBC)
- Phase I/II (n = 495 and 144 patients with TNBC)
Patients with TNBC or other solid metastatic cancer refractory to standard therapy

ASCENT (n = 529):
- TNBC without BM (n = 468):
- SG: 235 patients
- CT: 233 patients</td><td colspan="2">IMMU-132:
• Phase I: 8-10 mg/kg is the maximum tolerated dose after single infusion and multiple repeat cycles.
• Phase I/II (n = 69): The 10 mg/kg posology is ideal as it offers a higher efficacy and safety profile than the 8 mg/kg dose. The latter has a higher incidence of AEs and lower efficacy in comparison.
• The OS half-life at the 10 mg/kg dose is 11.7 hours.
• The SN38 of SG is bound to IgG in more than 95% of the total cytotoxic load, prolonging its action in the body by delaying its metabolism. This increases its level of efficacy, although it may increase the incidence of AEs.
• Dose adjustment or contraindication of SG is recommended in moderate/severe hepatic impairment.
• The different haplotypes involved in the UGT1A1 enzyme alteration imply an increased risk of developing AEs compared to the rest of the population.
• The homozygous haplotype has a higher risk of developing neutropenia.
• All haplotypes have the same risk of developing diarrhea and all three had to discontinue treatment at some point.
• - There are no recommendations for screening for UGT1A1 alterations in patients receiving SG.</td><td>It summarizes all phases of the clinical trials to date, reiterates that the data are truthful and objective, and adds some additional data. It examines the initial approval process by the FDA to conduct a Phase III clinical trial. It also describes the efficacy and safety data that supported approval of the drug for use in the clinical setting.</td></tr>
</table>

	TNBC with CM (n = 61)	**ASCENT:**<br>• TNBC without CM:<br>o Median PFS: SG (5.6 months); CT (1.7 months)<br>o Median OS: SG (12.1 months); CT (6.7 months)<br>o ORT: SG (35%); CT (5%)<br>• TNBC with CM:<br>o Median PFS: SG (2.8 months); CT (1.6 months)<br>o ORT: SG (3%); CT (0%)<br>o CBR: SG (9.4%); CT (3.4%)<br>o Median OS: No improvement was seen in patients treated with SG compared to CT.<br>• The percentages of the most common AEs were higher in the SG group than in the CT group and were as follows<br>o Neutropenia: 63%.<br>o Diarrhea: 59%.<br>o Nausea: 57%.<br>o Alopecia: 46%.<br>• Prophylaxis with granulocyte-colony stimulating factor (G-CSF) is recommended in patients with neutropenia.	

PUBLICATION DETAILS				
**REFERENCE**	**AUTHORS**	**DATE**	**TYPE OF STUDY**	**CLINICAL TRIAL**
[31]	Spring LM, et al.	10.07.2021	Systematic Review	IMMU-132 and ASCENT

RESEARCH CONTENT			
**OBJECTIVES**	**SAMPLE**	**RESULTS**	**CONCLUSIONS**
Study the results of both clinical trials and evaluate the management of AEs and SG toxicities in both.<br>Also evaluate pharmacokinetics and effects in patients with UGT1A1 alterations.	It discusses several clinical trials, but does not provide sample data to reflect recommendations and management of AEs.<br>However, all subjects are patients with metastatic TNBC and refractory to more than two treatments.	**SG Pharmacokinetics:**<br>• The metabolite of SG, SN38 is metabolized through UGT1A1. Therefore, the presence of an altered haplotype implies reduced enzymatic activity and results in a slowed metabolism of the cytotoxic SN38.<br>• SN38 exposure in patients with altered UGT1A1 haplotypes is elevated.<br>• Incidence of grade 4 neutropenia:<br>○ Homozygotes (28*28): 26%.<br>○ Heterozygotes (1*28): 13%.<br>○ Wild type (1*1): 11%.<br>• Systematic screening for altered UGT1A1 haplotypes prior to initiation of SG treatment is not recommended. However, it is recommended that patients previously diagnosed with this condition be closely monitored during the trial.<br>**Prevention and treatment recommendations for AE's according to the FDA:**<br>• Nausea and vomiting: Premedicate with 5HT3 antagonists, dexamethasone, and NK1 antagonists. And outpatient prophylaxis with ondansetron or prochlorperazine.<br>• Acute diarrhea or cholinergic syndrome in the first 24 hours after infusion: intravenous atropine.	The pharmacokinetics of SG are mainly hepatic, so the adverse effect it may have in patients with altered UGT1A1 haplotypes, in addition to other diseases that interfere with hepatic metabolism, should be evaluated.<br>Regarding the management of AEs, there is no specific protocol, but the FDA has developed recommendations based on how the various clinical trials were conducted and the results they provided.

		• Chronic or late diarrhea: exclude infectious process and prescribe oral loperamide.<br>• Neutropenia or febrile neutropenia: G-CSF after the first infusion for grade 4 neutropenia lasting more than 7 days, grade 3 neutropenia or fever of 38.5º, neutropenia that delays dosing for more than 2-3 weeks, and neutropenia of any grade after the first infusion. They also recommend progressive dose reductions according to the number of recurrences of this AE.<br>• Hypersensitivity reactions: premedication with acetaminophen, anti-H1, anti-H2 and additional corticosteroids.<br>• Anemia and thrombocytopenia: No specific recommendations, but several articles comment on the contribution of blood transfusion in these cases.<br>• Skin rash: Topical corticosteroids, oral anti-H1, and referral to dermatology.<br>• Alopecia: Wigs or scalp cooling cap devices, although they are still under investigation.	

PUBLICATION DETAILS				
**REFERENCE**	**AUTHORS**	**DATE**	**TYPE OF STUDY**	**CLINICAL TRIAL**
[32]	Sathe AG, et al.	29.05.2022	Study cohort	IMMU-132 and ASCENT

RESEARCH CONTENT			
**OBJECTIVES**	**SAMPLE**	**RESULTS**	**CONCLUSIONS**
To evaluate the pharmacokinetics of SG.	(n = 529)<br><br>Patients with metastatic TNBC and other solid tumors refractory to prior therapy. These are patients from the IMMU-132 (n = 276) and ASCENT (n = 253) studies who received SG.	**Pharmacokinetics:**<br>• No accumulation of IgG-bound metabolite or free SN38 was observed after multiple dose cycles.<br>• No clinically relevant or significant effects were observed between the pharmacokinetics of the drug and the following pathologies<br>○ Mild/moderate RI<br>○ Age<br>○ Gender<br>○ Albumin level<br>○ Race<br>○ ECOG status<br>○ Solid epithelial tumor type<br>○ UGT1A1 Haplotype<br>○ Trop expression (+1, +2, +3)<br>• There is evidence that SG can be used in patients with chronic and severe RI.	The pharmacokinetics of SG is mostly composed of SN38 bound to an IgG Ac, so its elimination is slower.<br>This analysis supports the clinical regimen of 10 mg/kg.<br>No need to adjust the dose according to pathology was observed, but patients with liver disease should be further studied.

<table>
<tr><th colspan="5">PUBLICATION DETAILS</th></tr>
<tr><th>REFERENCE</th><th>AUTHORS</th><th>DATE</th><th>TYPE OF STUDY</th><th>CLINICAL TRIAL</th></tr>
<tr><td>[33]</td><td>Ocean AJ, et al.</td><td>30.05.2017</td><td>Phase I/II clinical trial</td><td>IMMU-132</td></tr>
<tr><th colspan="5">RESEARCH CONTENT</th></tr>
<tr><th>OBJECTIVES</th><th>SAMPLE</th><th colspan="2">RESULTS</th><th>CONCLUSIONS</th></tr>
<tr><td>To evaluate the pharmacokinetics, efficacy and determine the safety of SG as monotherapy at doses of 8 and 10 mg/kg. To establish a standard dosing regimen.</td><td>(n = 178)

Patients with solid epithelial cancer refractory to more than three prior therapies. Of which (n = 53) had TNBC.

Divided into two groups:
- 8 mg/kg (n = 81)
- 10 mg/kg (n = 97)</td><td colspan="2">• This IMMU-132 trial resulted in FDA approval of the drug.
Pharmacokinetics:
• IgG-bound SN38 (>95%) remains in patient serum for longer periods (>24 hours). In contrast to free SN38, which is cleared more rapidly.
Efficacy:
• ORR: 8 mg/kg (10%); 10 mg/kg (22%)
• CBR: Was higher in the 10 mg/kg group
AEs:
• No anti-SG response was detected in serum after each infusion, indicating that the organism is not developing resistance to the drug.
• Dose reductions due to AEs: 8 mg/kg (15 patients); 10 mg/kg (26 patients).
• Study discontinuations due to AEs: 8 mg/kg (7 patients); 10 mg/kg (10 patients)
• Incidence of AEs: 8 mg/kg (99%); 10 mg/kg (92%)
• 10 mg/kg had a higher incidence of grade 3 AEs.
• There was no significant difference between grade 1-2 AEs and doses of 8 and 10 mg/kg.
• Grade >3 neutropenia:
○ After 1st dose: 8 mg/kg (21%); 10 mg/kg (47%).</td><td>The 10 mg/kg dose had an overall encouraging response in patients in terms of drug efficacy.
It is concluded that the SG regimen in monotherapy that offers objective responses, in addition to a good therapeutic index, is 10 mg/kg intravenously, on days 1 and 8 of 21-day cycles.</td></tr>
</table>

- After 1st-2nd doses: 8 mg/kg (50%); 10 mg/kg (63%)
- After >3rd dose: 8 mg/kg (50%); 10 mg/kg (42%)

AEs in patients with UGT1A1 enzyme alteration (n = 146):

	**Homozygous 28*28**	**Heterozygous 1*28**	**Wild type 1*1**
Sample	n = 19	n = 64	n = 63
Neutropenia	58%	39%	38%
Neutropenia after the 1st cycle	6 patients	11 patients	11 patients
Diarrhea	16%	8%	5%

- Neutropenia or diarrhea are more frequent and severe in patients with UGT1A1 haplotype alterations. However, their severity is not associated with doses of 8 or 10 mg/kg.

<table>
<tr><th colspan="5">PUBLICATION DETAILS</th></tr>
<tr><th>REFERENCE</th><th>AUTHORS</th><th>DATE</th><th>TYPE OF STUDY</th><th>CLINICAL TRIAL</th></tr>
<tr><td>[34]</td><td>Bardia A, et al.</td><td>14.03.2017</td><td>Phase I/II clinical trial</td><td>IMMU-132</td></tr>
<tr><th colspan="5">RESEARCH CONTENT</th></tr>
<tr><th>OBJECTIVES</th><th>SAMPLE</th><th colspan="2">RESULTS</th><th>CONCLUSIONS</th></tr>
<tr><td>Determine the efficacy of SG in monotherapy.
To assess safety, ORR, PFS and OS.</td><td>(n = 69)
Patients with metastatic TNBC who are refractory to more than one treatment prior to SG.</td><td colspan="2">Eficacia:
• Tumor burden reduction: 69.5%.
• ORR: 30% (13 patients with tumor size reduction >30% at first CT scan after 8 weeks of SG treatment)
• PR: 19 patients
• CR: 2 patients
• CBR: 46%.
• Median DOR: 8.9 months (95% CI 6.1 - 11.3)
• Median time to objective response 1.9 months
• Median PFS: 6 months (95% CI 5 - 7.3)
• Median OS 16.6 months (95% CI 11.1 - 20.6)
EAs grado >3 (41%):
• Neutropenia: 39%.
• Febrile neutropenia: 7%.
• Leukopenia: 16
• Anemia: 14%.
• Diarrhea: 13%
• Vomiting: 10%
• Thrombocytopenia: 3%.
• No SG-related deaths occurred
Trop 2 positive expression (n = 48):
• 42 patients had moderate and heavy staining. And they had a positive response</td><td>SG was well tolerated and induced early and durable objective responses in patients with metastatic TNBC who had previously received standard intensive chemotherapy. Trop 2 expression and its relationship with SG outcomes, in addition to being a possible prognostic marker of the disease, needs to be studied in depth.</td></tr>
</table>

		• 6 patients had weak or no staining. They had a positive response to treatment, but it was less favorable than that of patients with higher Ag expression.<br>• A higher PFS trend was observed in patients with moderate/strong Trop 2 expression compared to weak expression (7.1 months vs. 3.1 months).<br>**ELISA:** No anti-SN38 or antihRS7 Ac was detected. Therefore, the patients did not develop tolerance or resistance to the SG drug despite multiple cycles of treatment..<br>**BRCA 1 (n = 43):**<br>• RP: 7 patients<br>• RC: 2 patients<br>• Stable disease: 15 patients<br>• Progressive disease: 12 patients	

<table>
<tr><th colspan="5">PUBLICATION DETAILS</th></tr>
<tr><th>REFERENCE</th><th>AUTHORS</th><th>DATE</th><th>TYPE OF STUDY</th><th>CLINICAL TRIAL</th></tr>
<tr><td>[35]</td><td>Shastry M, et al</td><td>18.10.2022</td><td>Systematic Review</td><td>IMMU-132 and ASCENT</td></tr>
<tr><th colspan="5">RESEARCH CONTENT</th></tr>
<tr><th>OBJECTIVES</th><th>SAMPLE</th><th colspan="2">RESULTS</th><th>CONCLUSIONS</th></tr>
<tr><td>Investigation of Trop 2 as a therapeutic target and even as a prognostic marker of the disease.</td><td>Patients with metastatic TNBC who are refractory to prior standard therapy.</td><td colspan="2">• The higher the expression of Trop 2, the worse the OS rate, but it does not interfere with PFS and ORR results, and even potentiates them.
• An improvement in PFS, OS and ORR is demonstrated in patients with TNBC treated with SG compared to the results offered by CT at the choice of the treating physician.
• The most common AEs in patients with Trop 2 overexpression were neutropenia and diarrhea.</td><td>Given the results provided by SG, it is expected that future clinical trials will be able to evaluate the potential of SG as a 1st and 2nd line therapy in the treatment of metastatic TNBC and even in earlier stages of the disease.</td></tr>
</table>

<table>
<tr><th colspan="5">PUBLICATION DETAILS</th></tr>
<tr><th>REFERENCE</th><th>AUTHORS</th><th>DATE</th><th>TYPE OF STUDY</th><th>CLINICAL TRIAL</th></tr>
<tr><td>[36]</td><td>Wahby S, et al.</td><td>01.04.2021</td><td>Systematic Review</td><td>IMMU-132</td></tr>
<tr><th colspan="5">RESEARCH CONTENT</th></tr>
<tr><th>OBJECTIVES</th><th>SAMPLE</th><th colspan="2">RESULTS</th><th>CONCLUSIONS</th></tr>
<tr><td>Safety evaluation of SG in the clinical trials performed which led to accelerated approval of the SG drug by the FDA.</td><td>(n = 408)
Patients with metastatic and advanced solid epithelial cancer who had received more than three prior standard therapies, including 108 patients with TNBC.</td><td colspan="2">• On April 22, 2020, the FDA granted SG accelerated approval based on the results of the sample of 108 TNBC patients.
• This article describes the efficacy and AE results from the clinical trial. However, it also provides data not referenced in the study, such as
• Median DOR (n = 36): 7.7 months
○ DOR of >6 months but <12 months 55.6%
○ DOR of >12 months: 16.7%.
• - Median response time (n = 36): 2 months.
UGT1A1 evaluation (343 of 408 patients):
• Eighty-four percent of all patients in the sample had a UGT1A1 haplotype change.
• Grade 4 neutropenia:
○ 28*28 (39 patients): 26%.
○ 1*28 (155 patients): 13%
○ 1*1 (149 patients): 11%
• A patient with cancer and an altered UGT1A1 haplotype should be closely monitored during SG treatment in order to identify and manage AEs early so that they do not require treatment discontinuation.</td><td>SG efficacy results continue to be encouraging, leading to FDA approval of the drug.

The UGT1A1 haplotype alteration appears to be highly prevalent in patients with various types of epithelial solid tumors, so its presence in association with the incidence of AEs is a question mark in determining whether or not to screen for AEs when treated with SG. However, the FDA does not recommend routine screening.</td></tr>
</table>

<table>
<tr><th colspan="6">PUBLICATION DETAILS</th></tr>
<tr><th>REFERENCE</th><th>AUTHORS</th><th colspan="2">DATE</th><th>TYPE OF STUDY</th><th>CLINICAL TRIAL</th></tr>
<tr><td>[37]</td><td>Bardia A, et al.</td><td colspan="2">20.02.2019</td><td>Phase II clinical trial</td><td>IMMU-132</td></tr>
<tr><th colspan="6">RESEARCH CONTENT</th></tr>
<tr><th>OBJECTIVES</th><th>SAMPLE</th><th colspan="3">RESULTS</th><th>CONCLUSIONS</th></tr>
<tr><td>To evaluate the ORR, DOR, CBR, PFS and OS of SG in monotherapy. In addition, to study the incidence of AEs on the sample.</td><td>(n = 108)
Patients with metastatic TNBC who have failed a median of three prior standard therapies.</td><td colspan="3">• - This clinical trial resulted in FDA approval of the SG.
Efficacy:
• ORR: 36%.
• PR: 33 patients
• CR: 3 patients
• CBR: 45.4%.
• Stable disease >6 months: 40 patients (37%)
• Median DOR (n = 36) 7.7 months (95% CI 4.9 - 10.8)
  ○ DOR of >6 months but <12 months: 55.6%.
  ○ DOR of >12 months: 16.7%.
• Median time to response (n = 36) 2 months (95% CI 1.6 - 13.5)
• Median PFS: 5.5 months (95% CI 4.1 - 6.3)
• Median OS: 13 months (95% CI 11.2 - 13.7)
• Probability of response at 6 months: 59.7%.
• Probability of response at 12 months: 27%.
  ○ 6 patients had a durable response >12 months (95% CI 12.7 - 30.4).
• When evaluating the various patient subgroups, no significant differences in response rates were observed based on age, presence of metastatic disease, number of prior therapies or presence or absence of visceral metastases.
AEs:</td><td>OS is associated with durable objective and positive responses in patients with TNBC refractory to previous standard therapies. Myelotoxic effects were the most common adverse events.</td></tr>
</table>

		• Treatment discontinuation: 100 patients (86 due to disease progression)<br>• Dose reductions: 33% due to neutropenia<br>• Deaths: 77 patients none due to treatment AEs<br>○ 4 deaths due to treatment-emergent AEs attributed to disease progression >30 days after last infusion.<br>○ No deaths related to SG AEs were reported.<br>• Most common AEs (>25%): nausea, diarrhea, fatigue, neutropenia, anemia, alopecia, constipation, rash, decreased appetite, and abdominal pain.<br>• AE grade >3 (>5%): Neutropenia and anemia.<br>• No grade >3 peripheral neuropathy was observed.<br>• Diarrhea (62%): G1 (78%); G2 (14%); G>3 (8%).	

<table>
<tr><th colspan="5">PUBLICATION DETAILS</th></tr>
<tr><th>REFERENCE</th><th>AUTHORS</th><th>DATE</th><th>TYPE OF STUDY</th><th>CLINICAL TRIAL</th></tr>
<tr><td>[38]</td><td>Bardia A, et al.</td><td>16.03.2021</td><td>Phase I/II clinical trial</td><td>IMMU-132</td></tr>
<tr><th colspan="5">RESEARCH CONTENT</th></tr>
<tr><th>OBJECTIVES</th><th>SAMPLE</th><th colspan="2">RESULTS</th><th>CONCLUSIONS</th></tr>
<tr><td>Evaluation of the efficacy of SG by ORR, DOR, CBR, PFS and OS of the SG drug. In addition to the incidence of AEs</td><td>(n = 495)
Patients with advanced solid epithelial cancer who had received more than two prior therapies.
(n = 144)
Patients with TNBC.

98.6% of patients had stage IV disease.</td><td colspan="2">• This clinical trial resulted in accelerated approval of the drug by the FDA.
Efficacy (n = 144 patients with TNBC):
• ORR: 33.3%.
• Median ORR: 7.7 months
AEs (n = 495):
• Interruption of treatment: 483 patients (97.3%)
  ○ Progressive disease: 335 patients (67.7%)
  ○ Adverse event: 41 patients (8.3%)
  ○ 1 death due to pneumonia secondary to bronchial aspiration.
• Most common AEs
  ○ Nausea: 62.6%.
  ○ Neutropenia: 57.8%.
  ○ Diarrhea: 56.2%.
  ○ Fatigue: 48.3%
  ○ Alopecia: 40.4%
  ○ Febrile neutropenia: 5.5%.
• Grade >3 AEs (n = 295)
  ○ Nausea: 3.6%
  ○ Diarrhea: 7.9%
  ○ Neutropenia: 28.9%
  ○ Febrile neutropenia: 4.2% o Anemia: 10.3
  ○ Anemia: 10.3%.</td><td>SG demonstrated a toxicity profile consistent with previous reports. Of note was the safety and improved efficacy of the 10 mg/kg dose. In addition, efficacy was observed in multiple cancer cohorts, validating Trop 2 as a broad target in epithelial solid tumors.</td></tr>
</table>

UGT1A1 (n = 403):

	**Homozygous 28*28**	**Heterozygous 1*28**	**Wild type 1*1**
Neutropenia	60.9%	38.8%	33.3%
Diarrhea	60.9%	51.7%	54.8%
Anemia	50%	31.7%	37.3%
Treatment interruption	97.8%	96.7%	98.3%

- Homozygous patients are more prone to anemia and neutropenia. Diarrhea had a similar incidence in all three haplotypes. And treatment discontinuation occurred in all patients of each haplotype (temporality is not determined).

Drug discontinuation:

- 8 mg/kg: 56.8% of the 81 patients who received this dose in the clinical trial.
- 10 mg/kg: 49.5% of the 402 patients who received this dose.
- 12 mg/kg: 88.9% of the 9 patients who received this dose in the clinical trial.
- 18 mg/kg: 100% of the 3 patients who received this dose in the clinical trial.

<table>
<tr><th colspan="5">PUBLICATION DETAILS</th></tr>
<tr><th>REFERENCE</th><th>AUTHORS</th><th>DATE</th><th>TYPE OF STUDY</th><th>CLINICAL TRIAL</th></tr>
<tr><td>[39]</td><td>Schlam I, et al</td><td>04.10.2023</td><td>Systematic Review</td><td>IMMU-132 and ASCENT</td></tr>
<tr><th colspan="5">RESEARCH CONTENT</th></tr>
<tr><th>OBJECTIVES</th><th>SAMPLE</th><th colspan="2">RESULTS</th><th>CONCLUSIONS</th></tr>
<tr><td>Evaluation and management of AEs and potential SG toxicities.</td><td>Patients with metastatic TNBC who are refractory to prior standard therapies.</td><td colspan="2">FDA Recommendations for AE Prevention (ASCENT):
• Neutropenia (63 – 72%):
○ Mean time to presentation G>3: 21 days after SG infusion.
○ Delays in the following doses: SG (46%); CT (21%).
○ Reductions in the following doses: SG (11%); CT (19%)
○ Standard G-CSF prophylaxis: SG (29%); CT (10%)
○ Approximate protocol: Filgrastim for 3-4 days after day one and then pegfilgrastim after day eight.
○ Primary prophylaxis is not given, only secondary and at the discretion of the treating physician, as there is no standard protocol for management.
• Febrile neutropenia (6%):
○ Considered an oncologic emergency
○ Low-risk patient:
▪ Initial dose of IV antibiotics.
▪ If stable after 4 hours of infusion, oral treatment may be considered.
○ High risk patient:</td><td>The FDA's recommendations for the management and prevention of AEs suggest some ideas for dealing with the various AEs produced by SG. However, there is no standard protocol to follow, so it is often up to the treating physician to prescribe different treatments at his or her discretion.

Although individuals with selected UGT1A1 polymorphisms may be at increased risk of toxicity, it is unclear whether pretreatment testing is cost-effective and is not the standard of care. However, UGT inhibitors and inducers should be used with caution in combination</td></tr>
</table>

		▪ Urgent broad-spectrum IV antibiotics and hospitalization of the patient.<br>▪ After recovery, SG may be continued with dose reduction or G-CSF support.<br>▪ This AE is not a cause for study discontinuation.<br>• **Anemia (34 – 50%):**<br>○ Symptomatic patients or patients with Hb <7 g/dL:<br>▪ Prophylaxis with red blood cell transfusion.<br>○ Patients with severe or persistent anemia:<br>▪ Dose reduction or discontinuation<br>• **Nausea/Vomiting (60%):**<br>○ SG is considered by the NCCN to be a highly emetogenic agent.<br>○ Prophylaxis is recommended with 5HT3 antagonists (ondansetron, palonosetron), dexamethasone, NK1 antagonists, and olanzapine.<br>• **Diarrhea:**<br>○ Cholinergic syndrome in <24 hours after SG treatment: Intravenous atropine in future infusions until SG treatment is completed.<br>○ Late onset: Rule out infectious etiology, then prescribe loperamide, fluid and electrolyte replacement.<br>○ In addition to dose reductions based on event: 1st event (25% reduction), 2nd event (50%), and 3rd event (discontinue SG).<br>• **Alopecia:**	with SG, and testing may be considered for those with severe toxicities to determine the etiology and whether patients should continue SG treatment or receive irinotecan as a future line of therapy, especially as SG expands beyond breast cancer.

		○ Cooling caps during treatment infusion: It has been shown to be an effective method of reducing the rate of alopecia by 50% in patients treated with standard CT (anthracyclines and taxanes). Efficacy in patients treated with ADC requires further study.<br>**UGT1A1:**<br>• FDA Requirements for Irinotecan Treatment:<br>○ UGT1A1 haplotypes have been associated with increased toxicity of irinotecan.<br>○ The FDA recommended in 2005 that patients with haplotype 28*28 should receive a reduced dose of the drug.<br>○ Systematic screening of patients receiving irinotecan was not considered cost-effective.	

<table>
<tr><th colspan="5">PUBLICATION DETAILS</th></tr>
<tr><th>REFERENCE</th><th>AUTHORS</th><th>DATE</th><th>TYPE OF STUDY</th><th>CLINICAL TRIAL</th></tr>
<tr><td>[40]</td><td>Bardia A, et al.</td><td>22.04.2021</td><td>Phase III clinical trial</td><td>ASCENT</td></tr>
<tr><th colspan="5">RESEARCH CONTENT</th></tr>
<tr><th>OBJECTIVES</th><th>SAMPLE</th><th colspan="2">RESULTS</th><th>CONCLUSIONS</th></tr>
<tr>
<td>To compare efficacy outcomes and incidence of AEs in patients with non-BM metastatic TNBC treated with SG versus CT.</td>
<td>(n = 468) and (n = 61) Patients with recurrent and unresectable advanced metastatic TNBC. Without and with BM, respectively. Previously treated with more than two prior standard therapies (100% were treated with taxanes).
- SG Group (n = 235)
- CT Group (n = 233)

CT (Eribulin 54%, Vinorelbine 20%, Capecitabine 13%, Gemtacibine 12%)

No change of group from TC to SG was</td>
<td colspan="2">Efficacy in the 468 non-BM TNBC patients:
<table>
<tr><th></th><th>SG</th><th>CT</th></tr>
<tr><td>ORR</td><td>35%</td><td>5%</td></tr>
<tr><td>CBR</td><td>45%</td><td>9%</td></tr>
<tr><td>mPFS</td><td>5.6 months
(CI 95% 4.3 – 6.3)</td><td>1.7 months
(CI 95% 1.5 – 2.6)</td></tr>
<tr><td>mOS</td><td>12.1 months
(CI 95% 10.7 – 14)</td><td>6.7 months
(CI 95% 5.8 – 7.7)</td></tr>
<tr><td>mDOR</td><td>6.3 months</td><td>3.6 months</td></tr>
</table>
Efficacy in the 61 TNBC patients with BM:
<table>
<tr><th></th><th>SG</th><th>CT</th></tr>
<tr><td>mPFS</td><td>4.8 months
(CI 95% 4.1 – 5.8)</td><td>1.7 months
(CI 95% 1.5 – 2.5)</td></tr>
<tr><td>mOS</td><td>11.8 months
(CI 95% 10.5 – 13.8)</td><td>6.9 months
(CI 95% 5.9 – 7.7)</td></tr>
</table>
Most frequent AE's:
<table>
<tr><th></th><th>SG</th><th>CT</th></tr>
<tr><td>Neutropenia</td><td>63%</td><td>43%</td></tr>
<tr><td>Diarrhea</td><td>59%</td><td>12%</td></tr>
<tr><td>Nausea</td><td>57%</td><td>26%</td></tr>
<tr><td>Alopecia</td><td>46%</td><td>16%</td></tr>
<tr><td>Fatigue</td><td>45%</td><td>30%</td></tr>
<tr><td>Anemia</td><td>34%</td><td>24%</td></tr>
</table>
</td>
<td>SG showed significantly longer PFS and OS compared to CT in patients with metastatic TNBC. A higher incidence of AEs such as neutropenia, anemia and diarrhea was observed with SG.

The benefit of SG over CT in PFS was consistently observed in all pre-specified subgroups, including patients aged >65 years, those with >3 prior therapies, prior use of PD-1/PD-L1 inhibitors, TNBC at initial diagnosis with or without liver metastases.</td>
</tr>
</table>

<table>
<tr>
<td></td>
<td>allowed in case of progression.</td>
<td>
AE grade >4:
<table>
<tr><th></th><th>SG</th><th>CT</th></tr>
<tr><td>Neutropenia</td><td>51%</td><td>33%</td></tr>
<tr><td>Leukopenia</td><td>10%</td><td>5%</td></tr>
<tr><td>Diarrhea</td><td>10%</td><td><1%</td></tr>
<tr><td>Anemia</td><td>8%</td><td>5%</td></tr>
<tr><td>Febrile Neutropenia</td><td>6%</td><td>2%</td></tr>
</table>

Grade 4: Not seen in any group.
At the time of data cutoff, 15 patients in the SG group continued treatment because they did not have disease progression. In the CT arm, all patients progressed and none continued treatment.
Discontinuation due to toxicity: SG (5%); CT (5%).
Deaths:

SG: 3 deaths, none related to the drug but to disease progression (2 due to respiratory failure and 1 due to obstructive pneumonia).
CT: 3 deaths, one of which was attributed to treatment-emergent neutropenic sepsis.

</td>
<td></td>
</tr>
</table>

PUBLICATION DETAILS				
**REFERENCE**	**AUTHORS**	**DATE**	**TYPE OF STUDY**	**CLINICAL TRIAL**
[41]	O'Shaughnessy J, et al.	28.05.2021	Systematic Review	ASCENT

RESEARCH CONTENT			
**OBJECTIVES**	**SAMPLE**	**RESULTS**	**CONCLUSIONS**
Details the results of the TC group according to each type of treatment they used.<br><br>(Eribulin 54%, Vinorelbine 20%, Capecitabine 13%, Gemtacibine 12%)	(n = 529)<br>Patients with refractory metastatic TNBC, with and without BM.<br><br>- With BM: 61 patients<br>- Without BM: 468 patients<br>- SG: 235<br>- CT: 233<br><br>Of the 233 treated with CT:<br>- E: 126 patients<br>- V: 47 patients<br>- C: 31 patients<br>- G: 29 patients	See results below	The efficacy advantage of SG over CT in patients with TNBC was maintained when each CT agent was evaluated individually.

Efficacy:

	**SG**	**CT**	**E**	**V**	**C**	**G**
ORR	35%	5%	5%	4%	6%	3%
CBR	45%	9%	8%	6%	10%	14%
mPFS	5.6m	1.7m	2.1m	1.6m	1.6m	2.7m
mOS	12.1m	6.7m	6.9m	5.9m	5.2m	8.4m

AE grade >3 with Eribulin vs SG:

	**SG**	**E**
Neutropenia	51%	30%
Leukopenia	10%	5%
Fatigue	5%	3%
Anemia	8%	2%
Peripheral neuropathy	0%	2%

One treatment-emergent death in the TC (eribulin) group and none in the SG group. The cause was neutropenic sepsis secondary to chemotherapy-induced myelosuppression.

PUBLICATION DETAILS				
**REFERENCE**	**AUTHORS**	**DATE**	**TYPE OF STUDY**	**CLINICAL TRIAL**
[42]	Gilead Sciences	15.06.2022	Phase III clinical trial	ASCENT

RESEARCH CONTENT			
**OBJECTIVES**	**SAMPLE**	**RESULTS**	**CONCLUSIONS**
To compare the efficacy of SG with CT as measured by independently reviewed PFS, OS and ORR. in participants with metastatic TNBC treated with at least two systemic CT regimens for unresectable, locally advanced or metastatic disease, and no BM at baseline.	(n = 529)<br>Patients with metastatic TNBC treated with at least two prior systemic CT regimens for unresectable, locally advanced, non-BM disease at baseline.<br><br>These treatments were administered:<br>- SG<br>- Eribulin<br>- Capecitabine<br>- Gemcitabine<br>- Vinorelbine	**Inclusion criteria:**<br>• Histologically or cytologically confirmed TNBC based on the most recent analyzed biopsy or other pathologic specimen.<br>• Refractory or relapsed after at least two previous standard therapeutic regimens for metastatic TNBC.<br>• Prior exposure to a taxane in a localized or advanced/metastatic setting.<br>• Eligible for one of the listed CT options such as eribulin, capecitabine, gemcitabine or vinorelbine as assessed by the investigator.<br>• Eastern Cooperative Oncology Cooperative Group (ECOG) performance score of 0 or 1.<br>• CT or MRI measurable disease according to Response Evaluation Criteria in Solid Tumors version 1.1 (RECIST 1.1). Bone-only disease is not allowed.<br>• At least 2 weeks after previous anticancer treatment (CT, Hormone Therapy, RT and/or major surgery) and recovered from all acute toxicities of Grade 1 or less (except alopecia and peripheral neuropathy).<br>• At least 2 weeks after high doses of systemic corticosteroids (however, low doses of corticosteroids <20 mg prednisone or equivalent per day are allowed as long as the dose is stable for 4 weeks).	This is the clinical trial technical file that describes the inclusion protocol, treatment groups, and patients enrolled.

		• Adequate hematology without continuous transfusion support (hemoglobin > 9 g/dL, absolute neutrophil count (ANC) > 1500 per mm^3, platelets > 100,000 per mm^3).<br>• Adequate renal and hepatic function (creatinine clearance [CrCL] > 60 mL/min, bilirubin ≤ 1.5 times the institutional upper normal limit [IUNL], aspartate aminotransferase [AST] and alanine aminotransferase [ALT] ≤ 2.5 times the IUNL or ≤ 5 times the IUNL if liver metastases are known, and serum albumin ≥3 g/dl).<br>• Recovered from all toxicities to grade 1 or less according to the National Cancer Institute Common Terminology Criteria for Adverse Events (NCI CTCAE) v4.03 (except alopecia or peripheral neuropathy, which may be grade 2 or less) at the time of randomization. Participants with grade 2 neuropathy are eligible but may not receive vinorelbine as a CT.<br>• Participants with non-progressive BM treated and off high-dose steroids (>20 mg prednisone or equivalent) for at least 4 weeks are eligible.	

<table>
<tr><th colspan="5">PUBLICATION DETAILS</th></tr>
<tr><th>REFERENCE</th><th>AUTHORS</th><th>DATE</th><th>TYPE OF STUDY</th><th>CLINICAL TRIAL</th></tr>
<tr><td>[43]</td><td>Weide R, et al.</td><td>28.02.2023</td><td>Systematic Review</td><td>ASCENT</td></tr>
<tr><th colspan="5">RESEARCH CONTENT</th></tr>
<tr><th>OBJECTIVES</th><th>SAMPLE</th><th colspan="2">RESULTS</th><th>CONCLUSIONS</th></tr>
<tr><td>Efficacy analysis based on ORR, PFS and OS, in addition to assessing the incidence and severity of the various AEs presented by the SG drug.</td><td>(n = 529)
Patients with recurrent advanced metastatic TNBC and with/without BM. Previously treated with more than two prior lines of standard therapy.

TNBC without BM:
- SG Group (n = 235)
- CT Group (n = 233)</td><td colspan="2">Efficacy in the 468 TNBC patients without BM:
<table>
<tr><th></th><th>SG</th><th>CT</th></tr>
<tr><td>mPFS</td><td>5.6 months
(CI 95% 4.3 – 6.3)</td><td>1.7 months
(CI 95% 1.5 – 2.6)</td></tr>
<tr><td>mOS</td><td>12.1 months
(CI 95% 10.7 – 14)</td><td>6.7 months
(CI 95% 5.8 – 7.7)</td></tr>
</table>
Safety population (n = 482) (Patients who received at least one dose of either CT or SG):

SG: 258 patients
CT: 224 patients
AE grade >3:

<table>
<tr><th></th><th>SG</th><th>CT</th></tr>
<tr><td>Neutropenia</td><td>52%</td><td>33%</td></tr>
<tr><td>Diarrhea</td><td>11%</td><td>0.4%</td></tr>
<tr><td>Anemia</td><td>8%</td><td>5%</td></tr>
<tr><td>Febrile neutropenia</td><td>6%</td><td>2%</td></tr>
</table>

Quality of Life: SG demonstrated a significant clinical benefit in health-related quality of life (HRQoL) compared to TC.
</td><td>The endpoint blockade analysis of the ASCENT database confirms superior survival outcomes for SG compared to single-agent chemotherapy, with a manageable safety profile and improved quality of life for patients with TNBC in the second-line setting or beyond. These results support SG as an effective treatment option for this patient population.</td></tr>
</table>

PUBLICATION DETAILS				
**REFERENCE**	**AUTHORS**	**DATE**	**TYPE OF STUDY**	**CLINICAL TRIAL**
[44]	Carey LA, et al.	09.06.2021	Systematic Review	ASCENT

RESEARCH CONTENT

OBJECTIVES

Evaluate efficacy, incidence of adverse events, and details not described in the clinical trial [43].

SAMPLE

(n = 529)

Patients with recurrent unresectable advanced metastatic TNBC. With and without BM. Previously treated with more than two prior lines of standard therapy.

Patients with initial diagnosis of TNBC:

- SG: 79%
- CT: 84%

Patients without initial diagnosis of TNBC (had other previous cancer):

- SG: 21%
- CT: 16%

RESULTS

The previous treatments received in each group are described:

	SG	CT
Taxanes	100%	100%
Cyclophosphamide	91%	97%
Paclitaxel	85%	91%
Carboplatin	58%	84%
Doxorrubicin	48%	50%
Epirubicin	39%	44%
Inh of checkpoints	9%	13%

ECOG functional status:

- Patients in the TC group had worse functional status than patients in the SG group (ECOG PS of 1, 69% vs. 48%).
- There were no significant differences in renal or hepatic function between treatment groups.

Percentage of patients receiving treatment (x):

	SG	CT
Adjuvant	61%	41%
Neoadjuvant	82%	91%

CONCLUSIONS

In this analysis, some differences in baseline patient and disease characteristics were observed between treatment arms, which may indicate differences in bulky or aggressive disease. No significant differences in renal or hepatic function were observed between the two groups. Overall, there was no clear evidence of substantial differences in bulky or aggressive disease at baseline between treatment arms that would require more active disease management.

<table>
<tr><th colspan="5">PUBLICATION DETAILS</th></tr>
<tr><th>REFERENCE</th><th>AUTHORS</th><th>DATE</th><th>TYPE OF STUDY</th><th>CLINICAL TRIAL</th></tr>
<tr><td>[45]</td><td>Rugo HS, et al.</td><td>29.03.2022</td><td>Phase III clinical trial</td><td>ASCENT</td></tr>
<tr><th colspan="5">RESEARCH CONTENT</th></tr>
<tr><th>OBJECTIVES</th><th>SAMPLE</th><th colspan="2">RESULTS</th><th>CONCLUSIONS</th></tr>
<tr>
<td>Safety analysis of the effect of SG in patients according to age and altered UGT1A1 haplotypes.</td>
<td>(n = 529)
(n = 468) and (n = 61)
Patients with recurrent and unresectable advanced metastatic TNBC. With or without BM. Previously treated with more than two prior standard of care regimens.

- SG group (n=235)
- CT group (n=233)</td>
<td colspan="2">AE Neutropenia:
<table>
<tr><th></th><th>SG</th><th>CT</th></tr>
<tr><td>Time to onset of 1st G event>3 after treatment</td><td>21 days</td><td>14 days</td></tr>
<tr><td>Time to duration of G>3 episodes</td><td>6 days</td><td>6.5 days</td></tr>
<tr><td>Treatment-emergent neutropenia</td><td>63%
G>3 51%</td><td>43%
G>3: 33%</td></tr>
<tr><td>Febrile neutropenia</td><td>4%</td><td>0.4%</td></tr>
<tr><td>Use of G-CSF</td><td>49%</td><td>23%</td></tr>
<tr><td>Dose interruption</td><td>46%</td><td>21%</td></tr>
<tr><td>Dose reduction</td><td>11%</td><td>19%</td></tr>
</table>
AE Diarrhea:
<table>
<tr><th></th><th>SG</th><th>CT</th></tr>
<tr><td>Time to onset of 1st G event>3 after treatment</td><td>19 days</td><td>26.5 days</td></tr>
</table>
</td>
<td>SG demonstrated a manageable safety profile in patients with TNBC, including those ≥65 years of age (age is NOT a reason for older patients to respond better or worse and have more AEs than younger patients, however there were PFS, OS, and ORR results that showed the opposite in the study cohort [51]).</td>
</tr>
</table>

Time to duration of G>3 episodes	5 days	1 days
Treatment-emergent diarrhea	59%<br>G1 (30%)<br>G2 (19%)<br>G>3 (51%)<br>there was no G4-5	12%<br>G1 (7%)<br>G2 (5%)<br>G>3 (0.4%)<br>there was no G4-5
Atropine prophylaxis	10%	2%
Loperamide prophylaxis	55%	8%
Dose interruption	5%	0%
Dose reduction	5%	<1%

AE Nausea/Vomiting:

	**SG**	**CT**
Time to onset of first post-treatment event	8 days	8 days
Time to onset since start of treatment	24.5 days	17 days
Vomiting G>3	<3%	<3%
Prophylaxis (ondansetron, prochlorperazine)	86%	63%

Analysis of patients with UGT1A1 haplotypes (n = 250):

	**Homozygous 28*28**	**Heterozygous 1*28**	**Wild type 1*1**
Sample	34 patients	96 patients	113 patients
Neutropenia G>3	59%	47%	57%
Febrile N.	18%	5%	3%
Anemia	15%	6%	4%
Diarrhea	15%	9%	10%
Average dose reduction time	1.8 months	2.7 months	2.7 months
AEs that resulted in dose reduction	35%	19%	18%

- 28*28 had a slightly higher rate of G>3 neutropenia, febrile neutropenia, anemia, and diarrhea compared to other variants.
- Nausea, vomiting, constipation, fatigue, alopecia, and decreased appetite were not affected by UGT1A1 genotypic variant status.
- We remain in the debate as to whether or not we should screen for UGT1A1 genotyping in all pctes as they do in those with colorectal cancer and haplotype 28*28. The

		FDA requires a reduction in the initial dose of irinotecan in patients with colorectal cancer with known UGT1A1 28*28 status28 and the European Society for Medical Oncology guidelines recommend UGT1A1 genotyping in patients with metastatic colorectal cancer who are scheduled to receive irinotecan >180 mg/m 2 and are suspected of having UGT1A1 deficiency as evidenced by low levels of conjugated BB.	

PUBLICATION DETAILS				
**REFERENCE**	**AUTHORS**	**DATE**	**TYPE OF STUDY**	**CLINICAL TRIAL**
[46]	Dieras V., et al.	31.05.2021	Phase III clinical trial	ASCENT

RESEARCH CONTENT

OBJECTIVES

Study cohort of SG in TNBC patients with BM in the ASCENT clinical trial.

SAMPLE

(n = 61)

Patients with metastatic TNBC refractory to a median of 5 prior therapies and with BM.

BM had to be known and stable for >4 weeks as evidenced by MRI.

BMs had to be stable for >2 weeks since discontinuation of anticonvulsant and corticosteroid therapy.

Their analysis was separated from that of patients without BM to avoid bias in the evaluation of results.

RESULTS

Efficacy of patients with TNBC with BM:

	**SG**	**CT**
ORR	3%	0%
CBR	9.4%	3.4%
Stable dis.	47%	31%
mPFS	2.8 months	1.6 months
mOS	6.8 months	7.5 months
mDOR	6.7 months	-

AEs of any grade in patients with TNBC with BM:

	**SG**	**CT**
tiredness	63%	52%
diarrhea	50%	13%
Neutropenia	43%	35%
nausea	43%	26%
decreased appetite	30%	17%
decreased neutrophil count	33%	22%
anemia	23%	35%
alopecia	23%	13%
Constipation	23%	22%
Deaths	0	-

CONCLUSIONS

Interpretation of the data in this poor prognosis population is limited by the small sample size (61 TNBC + WITH BRAIN METS patients). In this exploratory analysis of patients with brain metastases from the ASCENT Phase 3 trial, SG was numerically superior to CT for ORR and PFS, but not for OS. The safety profile in both arms was similar to the population without brain metastases.

PUBLICATION DETAILS				
**REFERENCE**	**AUTHORS**	**DATE**	**TYPE OF STUDY**	**CLINICAL TRIAL**
[47]	Kalinsky K., et al.	28.05.2021	Phase III clinical trial	ASCENT

RESEARCH CONTENT

OBJECTIVES

SG study cohort in patients aged >65 years, >75 years, and <65 years in patients with non-BM TNBC from the ASCENT clinical trial.

SAMPLE

(n = 468)
SG >65y (n = 44)
CT >65y (n = 46)

Patients with metastatic and refractory TNBC, without BM, aged 65 years or older.

RESULTS

Efficacy in patients with TNBC without BM and age >65y:

	**Patients >65y (n = 90)**		**Patients <65y (n = 378)**	
Treatment	**SG**	**CT**	**SG**	**CT**
Sample	44	46	191	187
ORR	50%	0%	31%	6%
CBR	61%	9%	41%	9%
mPFS	7.1 m	2.4 m	4.6 m	1.7 m
mOS	15.3 m	8.2 m	11.2 m	6.6 m

Efficacy in patients with TNBC without BM and age >75y:

	**Patients >75y (n = 7)**
Treatment	**SG**
Sample	7 (All patients >75 years of age received SG)
RP	2 patients
Stable disease	4 patients
Stable dis. <6 meses	1 patient

CONCLUSIONS

Regardless of age, patients receiving SG had a significant survival benefit over CT with a tolerable safety profile. Close monitoring in addition to treatment and prevention of AEs will allow optimal therapeutic exposure to SG in elderly patients.

AE in patients with TNBC without BM and age >65y:

	SG	CT
Dose Reduction	>65a – 35%<br><65a – 19%	>65a – 33%<br><65a – 24%
Treatment interruption	>65a – 2%<br><65a – 5%	>65a – 2%<br><65a – 6%
Deaths	0	-

Causes of dose reduction in patients aged >65y:

	SG	CT
Febrile neutropenia	14%	25%
Fatigue	10%	4%
Diarrhea	6%	0%
Nausea	4%	0%

<table>
<tr><th colspan="5">PUBLICATION DETAILS</th></tr>
<tr><th>REFERENCE</th><th>AUTHORS</th><th>DATE</th><th>TYPE OF STUDY</th><th>CLINICAL TRIAL</th></tr>
<tr><td>[48]</td><td>Bardia A, et al.</td><td>02.06.2021</td><td>Phase III clinical trial</td><td>ASCENT</td></tr>
<tr><th colspan="5">RESEARCH CONTENT</th></tr>
<tr><th>OBJECTIVES</th><th>SAMPLE</th><th colspan="2">RESULTS</th><th>CONCLUSIONS</th></tr>
<tr>
<td>Study cohort evaluating Trop 2 TNBC tumor expression and BRCA 1/2 status and their relationship to clinical outcomes provided by SG in the ASCENT clinical trial.</td>
<td>Trop 2 + (n = 290)
- SG (n = 151)
○ +3: 56%
○ +2: 26%
○ +1: 18%
○ +0: 7 patients
- CT (n = 139)

BRCA 1/2 + (n = 292)
- SG (n = 149)
- CT (n = 143)

Patients with refractory metastatic TNBC and no BM..</td>
<td colspan="2">Efficacy of SG in patients according to Trop 2 expression.:
<table>
<tr><th></th><th colspan="3">SG</th><th colspan="3">CT</th></tr>
<tr><td>Expression</td><td>+3</td><td>+2</td><td>+1</td><td>+3</td><td>+2</td><td>+1</td></tr>
<tr><td>Sample</td><td>56%</td><td>26%</td><td>18%</td><td>-</td><td>-</td><td>-</td></tr>
<tr><td>ORR</td><td>44%</td><td>38%</td><td>22%</td><td>1%</td><td>11%</td><td>6%</td></tr>
<tr><td>mPFS</td><td>6.9m</td><td>5.6m</td><td>2.7m</td><td>2.5m</td><td>2.2m</td><td>1.6m</td></tr>
<tr><td>mOS</td><td>14.2m</td><td>14.9m</td><td>9.3m</td><td>6.9m</td><td>6.9m</td><td>7.6m</td></tr>
</table>
• PFS, OS and ORR were numerically higher with SG vs. TC in patients with high, medium and low Trop-2 expression.

Efficacy of SG in patients with BRCA 1/2 mutation.:
<table>
<tr><th></th><th colspan="2">SG</th><th colspan="2">CT</th></tr>
<tr><td>BRCA</td><td>+</td><td>-</td><td>+</td><td>-</td></tr>
<tr><td>ORR</td><td>19%</td><td>33%</td><td>6%</td><td>6%</td></tr>
<tr><td>mPFS</td><td>4.6m</td><td>4.9m</td><td>2.5m</td><td>1.6m</td></tr>
<tr><td>mOS</td><td>15.6m</td><td>10.9m</td><td>4.4m</td><td>7m</td></tr>
</table>
• Efficacy results were numerically superior with SG versus TC in patients with and without germline BRCA1/2 mutations.</td>
<td>Treatment with SG may be associated with improved clinical outcomes compared to standard TC in both BRCA mutation-positive and mutation-negative patients.
SG benefits previously treated TNBC patients expressing Trop-2 +3/+2 compared to standard TC and regardless of germline BRCA1/2 mutation status. The small number of patients with Trop-2 expression (+1 or +0) precludes definitive conclusions.</td>
</tr>
</table>

PUBLICATION DETAILS				
**REFERENCE**	**AUTHORS**	**DATE**	**TYPE OF STUDY**	**CLINICAL TRIAL**
[49]	O'Shaughnessy J, et al.	31.10.2021	Phase III clinical trial	ASCENT

RESEARCH CONTENT			
**OBJECTIVES**	**SAMPLE**	**RESULTS**	**CONCLUSIONS**
Cohort study of SG outcomes in patients with an initial diagnosis of TNBC compared to patients without an initial diagnosis of TNBC but with prior positive receptors (estrogen, progesterone, and Her2neu).	(n = 468)<br>Patients with TNBC without BM.<br>(n = 146)<br>Patients with TNBC without BM without initial diagnosis of TNBC. (Considered as patients with more aggressive disease).:<br>- SG (n = 70)<br>- CT (n = 76)<br><br>(n = 322)<br>TNBC patients without BM with an initial diagnosis of TNBC.<br>- SG (n = 155)<br>- CT (n = 157)	See results below	In the subset of patients without TNBC at initial diagnosis, there was a significant survival benefit with SG over CT, with a manageable safety profile. The results were similar to the overall population in the ASCENT trial, i.e., the results were similar to those of patients with a baseline diagnosis of TNBC.<br><br>SG demonstrated a similar clinical benefit in both patients with and without a baseline diagnosis of TNBC, when comparing these results to those of CT.

Efficacy of SG in patients with/without initial diagnosis of TNBC:

	**No initial diagnosis of TNBC (n = 146)**		**With initial diagnosis of TNBC (n = 322)**	
Treatment	**SG**	**CT**	**SG**	**CT**
Sample	70	76	155	157
ORR	31%	4%	36%	5%
RP/RC	30% / 1%	3% / 1%	31% / 5%	4% / 1%
mPFS	4.6 m	2.3 m	5.7 m	1.6 m
mOS	12.4 m	6.7 m	12.1 m	6.9 m

- Efficacy outcomes for patients with TNBC at initial diagnosis improved similarly to those without the diagnosis who were treated with SG vs. TC.

AEs of SG in pctes without initial diagnosis TNBC (n = 146):

	**SG**	**CT**
Neutropenia	59%	40%
Anemia	8%	7%
Diarrhea	7%	0%
Deaths	0%	-

<table>
<tr><th colspan="5">PUBLICATION DETAILS</th></tr>
<tr><th>REFERENCE</th><th>AUTHORS</th><th>DATE</th><th>TYPE OF STUDY</th><th>CLINICAL TRIAL</th></tr>
<tr><td>[50]</td><td>Carey LA, et al.</td><td>28.05.2021</td><td>Phase III clinical trial</td><td>ASCENT</td></tr>
<tr><th colspan="5">RESEARCH CONTENT</th></tr>
<tr><th>OBJECTIVES</th><th>SAMPLE</th><th colspan="2">RESULTS</th><th>CONCLUSIONS</th></tr>
<tr><td>SG study cohort in patients with TNBC who had relapsed in less than 12 months following neoadjuvant CT as standard therapy prior to enrollment in the study. And who subsequently received only one line of therapy in the metastatic setting.</td><td>(n = 65)
Patients with metastatic TNBC without BM who relapsed during their prior chemotherapy regimen before 12 months after their last infusion. Considering that these patients have more aggressive disease due to early relapse.
- SG (n = 33)
- CT (n = 32)</td><td colspan="2">Efficacy of SG in patients with TNBC who had disease recurrence within 12 months of their last standard therapy:
<table><tr><th></th><th>SG</th><th>CT</th></tr><tr><td>Sample</td><td>33 patients</td><td>32 patients</td></tr><tr><td>ORR</td><td>30%</td><td>3%</td></tr><tr><td>CBR</td><td>42%</td><td>6%</td></tr><tr><td>mPFS</td><td>5.7 months</td><td>1.5 months</td></tr><tr><td>mOS</td><td>10.9 months</td><td>4.9 months</td></tr><tr><td>mDOR</td><td>6.7 months</td><td>-</td></tr></table>• Efficacy results for this subgroup are similar to those of SG vs. TC in the general TNBC population without BM.

AEs in this cohort:
• The safety profile of SG in this subset of patients was consistent with previous reports.
• There were no deaths related to SG treatment.</td><td>Patients with TNBC who relapsed prior to 12 months after receiving standard chemotherapy indicated for TNBC and who subsequently received 1 prior line of therapy in the metastatic setting may represent a subset with more aggressive disease.
In this cohort, patients had superior outcomes with SG versus CT in the second-line metastatic setting, consistent with the benefit observed in the overall TNBC population without BM.</td></tr>
</table>

PUBLICATION DETAILS				
**REFERENCE**	**AUTHORS**	**DATE**	**TYPE OF STUDY**	**CLINICAL TRIAL**
[51]	Carey LA, et al.	15.02.2022	Phase III clinical trial	ASCENT

RESEARCH CONTENT

OBJECTIVES

SG study cohort in black patients, in whom TNBC and BRCA mutations are known to be more prevalent, in addition to being a typical diagnosis in young patients, with a high recurrence rate and worse outcomes.

SAMPLE

(n = 62)

Patients with metastatic TNBC with/without BM (n = 529). And of black race. Who had received a minimum of 2 previous chemotherapy treatments.

- SG (n = 28)
- CT (n = 34)

Incidence of BRCA + mutation in black patients (3%) in 2 patients out of the total sample of 62.

RESULTS

Efficacy of SG in TNBC patients with/without BM and black race:

	SG	CT
Sample	**28 patients**	**34 patients**
ORR	32%	6%
CBR	43%	15%
mPFS	5.4 months	2.2 months
mOS	13.8 months	8.5 months
mDOR	5.4 months	2.2 months

Most common AEs of grade >3 in this cohort:

	SG	CT
Neutropenia	48%	42%
Anemia	12%	6%
Leukopenia	8%	16%
Febrile N.	8%	3%
Suspended treatment	0%	3%
Deaths due to AEs	0%	-

CONCLUSIONS

Only 12% of patients in the ASCENT trial were identified as black (n=62). Patients in this cohort achieved a similar clinical benefit in SG compared to CT as observed in the overall study population. Along with a manageable safety profile. Thus, this cohort confirms that SG should be considered as a second-line treatment option in patients with TNBC.

PUBLICATION DETAILS				
**REFERENCE**	**AUTHORS**	**DATE**	**TYPE OF STUDY**	**CLINICAL TRIAL**
[52]	Loibl S., et al.	12.10.2021	Phase III clinical trial	ASCENT

RESEARCH CONTENT			
**OBJECTIVES**	**SAMPLE**	**RESULTS**	**CONCLUSIONS**
SG study cohort on health-related quality of life in patients treated with this drug.	(n = 419)<br>TNBC patients without BM who completed more than one EORTC QLQ-C30 questionnaire related to HRQoL. Completed at baseline, before each infusion, and at the end of the clinical trial..<br>- SG (n = 236)<br>- CT (n = 183)	**Results of the EORTC QLQ-C30 Questionnaire in Relation to HRQoL in TNBC Patients Treated with SG or TC:** (see table below)<br>• Diarrhea is the only symptomatic/clinical aspect where CT patients fared significantly better than SG patients.	In patients with TNBC, SG not only prolonged PFS and OS, but also improved HRQoL (Health-Related Quality of Life) compared to CT. Although diarrhea symptoms worsened with SG, this did not appear to translate into an adverse impact on patients' global health score or functioning.

	**SG**	**CT**
Overall health status	0.7	-3.4
Physical functioning	1.3	-4.4
Emotional sphere	3.3	-0.5
Fatigue SI	2.0	7.1
Pain SI	-8.9	-1.9
Dyspnea SI	-3.8	4
Insomnia SI	-4.7	0.3
Diarrhea SI	14.1	-1.3

PUBLICATION DETAILS					
**REFERENCE**	**AUTHORS**	**DATE**	**TYPE OF STUDY**		**CLINICAL TRIAL**
[53]	Sibylle SM, et al.	15.02.2022	Phase III clinical trial		ASCENT
**RESEARCH CONTENT**					
**OBJECTIVES**	**SAMPLE**	**RESULTS**			**CONCLUSIONS**
SG study cohort regarding health-related quality of life in patients treated with this drug.	(n = 419)<br>TNBC patients without BM who completed more than one EORTC QLQ-C30 HRQoL questionnaire.<br>Completed at baseline, before each infusion, and at the end of the clinical trial.<br><br>• SG (n = 236)<br>• CT (n = 183)	**CCVOE questionnaire responses:**<br>• At baseline: both treatment arms (SG and TC) had similar responses.<br>• Independent of clinical response**:**<br>○ SG patients had more favorable mean HRQoL changes than TC patients on most items of the EORTC QLQ-C30 questionnaire.<br>○ EXCEPT for nausea/vomiting and dizziness, where patients treated with TC had better HRQoL.<br><br>SG (n = 236) of which 82 patients (35%) had an ORR (RP/RC).<br><br>CT (n = 183) of which 11 patients (6%) had an ORR (PR/RC).			The analysis shows that regardless of ORR objective response status, SG showed a better trend in HRQoL changes than CT. Patients who achieved tumor response to SG may benefit more in terms of HRQoL. Although SG-treated patients reported higher rates of diarrhea, this did not adversely affect their quality of life or overall functioning.

PUBLICATION DETAILS				
**REFERENCE**	**AUTHORS**	**DATE**	**TYPE OF STUDY**	**CLINICAL TRIAL**
[54]	Loibl S, et al.	18.10.2022	Phase III clinical trial	ASCENT
**RESEARCH CONTENT**				
**OBJECTIVES**	**SAMPLE**	**RESULTS**		**CONCLUSIONS**
SG study cohort regarding health-related quality of life in patients treated with this drug.	(n = 419)<br>TNBC patients without BM who completed more than one EORTC QLQ-C30 questionnaire related to HRQoL. Completed at baseline, before each infusion, and at the end of the clinical trial.<br>- SG (n = 236)<br>- CT (n = 183)	• HRQoL outcomes after the first infusions, both SG and CT, were worse overall in both groups.<br>• Initially, worse scores were observed for the CT group in<br>○ Global health status<br>○ Quality of life<br>○ Insomnia<br>• The SG Group received:<br>○ Greater financial hardship.<br>○ Large changes for the better from baseline in global health status, quality of life, physical functioning, fatigue, and pain.<br>○ Few changes, all for the worse, from baseline in nausea, vomiting, and diarrhea. These AEs did not change in severity or incidence during the study, although they were from baseline. The use of prophylaxis is not reported.<br>○ The mean time to first clinical worsening was longer in the SG group than in the CT group. It was the same in terms of work functionality, fatigue and pain.		SG was associated with greater improvements and delayed worsening of quality of life scores compared to the CT group. This supports the favorable clinical profile of SG as a treatment for TNBC.

PUBLICATION DETAILS					
**REFERENCE**	**AUTHORS**	**DATE**	**TYPE OF STUDY**		**CLINICAL TRIAL**
[55]	Benedict A, et al.	31.01.2023	Phase III clinical trial		ASCENT
**RESEARCH CONTENT**					
**OBJECTIVES**	**SAMPLE**	**RESULTS**			**CONCLUSIONS**
SG study cohort to assess the health status of SG-treated TNBC patients at pre- and post-progression using the EORTC QLQ-C30 questionnaires.	(n = 479)<br>TNBC patients without BM who completed the EORTC QLQ-C30 for HRQoL at >1 observation.<br>(n = 411)<br>TNBC patients without BM who completed >1 EORTC QLQ-C30 questionnaire. These 411 patients were eligible for the benefit analysis of this cohort. Questionnaires were completed at baseline, before each cycle, and 3-4 weeks after completion of the clinical trial.	• The utility of SG was significantly higher compared to TC (+0.084).<br>• Pre-progression utility: SG (+0.710); CT (+0.626).<br>• Utility after progression: SG (+0.653); CT (+0.569).<br>• The UK physicians confirmed the plausibility of such a large difference and significant effect, which is consistent with the large responses observed in the trial with the SG drug.			Treatment with SG Provides Large Utility Gains vs. CT in Late Line TNBC, which is Transferred and Preserved After Progression.

<table>
<tr><th colspan="5">PUBLICATION DETAILS</th></tr>
<tr><th>REFERENCE</th><th>AUTHORS</th><th>DATE</th><th>TYPE OF STUDY</th><th>CLINICAL TRIAL</th></tr>
<tr><td>[56]</td><td>Cortés J, et al.</td><td>15.05.2022</td><td>Phase III clinical trial</td><td>ASCENT</td></tr>
</table>

<table>
<tr><th colspan="4">RESEARCH CONTENT</th></tr>
<tr><th>OBJECTIVES</th><th>SAMPLE</th><th>RESULTS</th><th>CONCLUSIONS</th></tr>
<tr>
<td>Analysis that examines the outcomes of TNBC patients treated with SG who subsequently progressed. In addition, it will attempt to objectify the SG of patients after discontinuation of SG due to progression.</td>
<td>(n = 222)
Patients with metastatic TNBC with/without BM who were treated with SG and discontinued treatment due to disease progression. That is, of the 267 patients who received SG out of the total sample of 529, 222 discontinued treatment due to disease progression.
• n = 163 patients received CT after SG progression.
• n = 59 patients did not receive CT or other treatment after progression.</td>
<td>Efficacy in TNBC patients treated with SG whose disease progressed after treatment.:
<table>
<tr><td></td><th colspan="5">SG</th></tr>
<tr><td>Post-progression therapy</td><th colspan="4">Yes
(n=163)</th><th>No
(n=59)</th></tr>
<tr><td>Treatment</td><td>ER</td><td>CAR</td><td>CAP</td><td>AT</td><td>-</td></tr>
<tr><td>Sample</td><td>70</td><td>34</td><td>34</td><td>15</td><td>59</td></tr>
<tr><td>Sample (%)</td><td>32%</td><td>15%</td><td>15%</td><td>7%</td><td>-</td></tr>
<tr><td rowspan="2">mOS from start of study</td><td>14.1m</td><td>13.6m</td><td>16.5m</td><td>14.9m</td><td rowspan="2">7.3 months</td></tr>
<tr><td colspan="4">Median of 13.4 months</td></tr>
<tr><td rowspan="2">mOS since end of study</td><td>8.4m</td><td>8.9m</td><td>8.6m</td><td>8.9m</td><td rowspan="2">2.0 months</td></tr>
<tr><td colspan="4">Median of 7.9 months</td></tr>
</table>
</td>
<td>In ASCENT, the majority of patients who discontinued SG due to disease progression were able to receive retreatment after progression. Patients who received post-progression therapy had a significantly better median OS than those who did not receive post-progression therapy. There was no significant difference in the chemotherapy they received. These results suggest that treatment with SG does not preclude the use of subsequent systemic therapy.</td>
</tr>
</table>

<table>
<tr><th colspan="5">PUBLICATION DETAILS</th></tr>
<tr><th>REFERENCE</th><th>AUTHORS</th><th>DATE</th><th>TYPE OF STUDY</th><th>CLINICAL TRIAL</th></tr>
<tr><td>[57]</td><td>Michaleas S., et al.</td><td>25.05.2022</td><td>European Medicines Agency Report</td><td>ASCENT</td></tr>
<tr><th colspan="5">RESEARCH CONTENT</th></tr>
<tr><th>OBJECTIVES</th><th>SAMPLE</th><th colspan="2">RESULTS</th><th>CONCLUSIONS</th></tr>
<tr><td>European Medicines Agency review of SG drug. Approval is based on efficacy and AE incidence results from the ASCENT clinical trial..</td><td>IMMU-132
ASCENT (n = 529)
Patients with metastatic TNBC with/without BM.</td><td colspan="2">SG received EU-wide marketing authorization on 22.11.2021.
Indications for treatment with SG in the EU include
Unresectable or metastatic TNBC with or without brain involvement, although outcomes were only evaluated in patients with non-ASCT TNBC.
SG prolongs PFS by 5 months and OS by 3 months compared to CT.
The most serious and frequent AEs with SG treatment were febrile neutropenia and diarrhea.
Clinical efficacy was demonstrated by reviewing the results of the IMMU-132 and ASCENT clinical trials.
Maximum tolerated dose in one infusion: 12 mg/kg
Maximum tolerated dose in multiple cycles and multiple infusions: 10 mg/kg and 8 mg/kg
ORR: 10mg/kg (22%); 8mg/kg (10%)
CBR: higher at 10 mg/kg than at 8 mg/kg
SG was associated with a statistically significant and clinically relevant improvement in PFS compared to CT in patients who had received two or more prior systemic therapies, including at least one in the advanced setting.</td><td>The clinical benefit of treatment with SG over CT is clear. These positive results are consistent regardless of BRCA mutation status.
The EMA Board concluded by consensus that the benefit/risk ratio of SG monotherapy is favorable for the treatment of adult patients with unresectable or metastatic TNBC who have received two or more prior systemic treatments, including at least one for advanced disease, and therefore recommended granting marketing authorization.</td></tr>
</table>

		• A clinically relevant effect was also observed with respect to the secondary endpoint of SG.<br>• SG toxicity was higher compared to standard TC, but was considered manageable with granulocyte colony-stimulating factor support and dose modifications.<br>• The most common AEs were hypersensitivity, diarrhea and serious infections secondary to neutropenia, while embryo-fetal toxicity was considered a potential risk.<br>• Information on the use of SG in patients with moderate or severe hepatic impairment and immunogenicity is lacking. However, at this time, it is not recommended that SG be administered to patients with severe hepatic pathology.	

PUBLICATION DETAILS				
**REFERENCE**	**AUTHORS**	**DATE**	**TYPE OF STUDY**	**CLINICAL TRIAL**
[58]	Xu B, et al.	15.05.2023	Phase IIb clinical trial	Conducted in China

RESEARCH CONTENT			
**OBJECTIVES**	**SAMPLE**	**RESULTS**	**CONCLUSIONS**
The objective is to evaluate the efficacy and safety of SG (10 mg/kg intravenously on days 1 and 8 in 21-day cycles) in 14 centers in mainland China.<br>China is known to have a high prevalence of BC, with an estimated 124,000 deaths in 2022.	(n = 80)<br>Patients with metastatic TNBC without BM and refractory to more than two prior standard therapies in unresectable disease. Patients with only one prior standard therapy, but whose disease had progressed <12 months since that treatment, were also included.	**SG Efficacy:**<br>• ORR: 38.8%<br>• RP: 36.3%<br>• RC: 2.5%<br>• Stable disease: 43.8%.<br>• CBR: 82.5%.<br>• Tumor shrinkage according to OS: 76.3% (61 patients)<br>• >30% tumor shrinkage by SG: 50% (40 patients)<br>• Median DOR: 5.59 months<br>• Time to onset of response: 1.51 months<br>• Median PFS: 5.55 months<br>• Median PFS at 6 months: 48.3% Median OS: 48.3% Median OS: 48.3%<br>• Median OS:<br>○ At 3 months: 93.8%<br>○ At 6 months: 82.5%<br>○ At 9 months: 68.0%<br>**AEs (n = 80):**<br>• The entire sample suffered from AE's<br>• Dose Delay: 35 patients (43.8%)<br>• Dose reduction: 12 patients (15%)<br>• SG interruption: 5 patients (6.3%)<br>• **Most common AEs**<br>○ Neutropenia: 68 patients (85%)	Overall, high post-treatment ORR was seen in all pre-specified subgroups, including those with lung/liver metastases.<br><br>The results appear to be consistent and similar to other clinical trials, both IMMU-132 and ASCENT.<br><br>A high incidence of AEs was observed in this clinical trial. As no prophylactic treatment or prevention of AEs was mentioned, it could be assumed that this is why the percentages are so high compared to ASCENT.

		○ Anemia: 66 patients (82.5%)<br>○ Leukopenia: 65 patients (81.3%)<br>○ Vomiting: 44 patients (55%)<br>○ Nausea: 40 patients (50%)<br>• **AEs G>3 related to SG:** 57 patients (71.3%)<br>○ Neutropenia: 50 patients (62.5%)<br>○ Leukopenia: 39 patients (48.8%)<br>○ Anemia: 17 patients (21.3%)<br>• **Deaths:** There is one possible treatment-emergent death due to shock secondary to diarrhea and myelosuppression (causes not specified, but may have been due to hypovolemic shock associated with neutropenic sepsis).<br>• The use of prophylaxis is not mentioned.<br>• **Metastasis sites:**<br>• Lymph nodes: 61.3%.<br>• Lungs: 52.5%.<br>• Bones: 33.8%.<br>• Liver: 30%.	

<table>
<tr><th colspan="5">PUBLICATION DETAILS</th></tr>
<tr><th>REFERENCE</th><th>AUTHORS</th><th>DATE</th><th>TYPE OF STUDY</th><th>CLINICAL TRIAL</th></tr>
<tr><td>[59]</td><td>Reinisch M, et al.</td><td>28.07.2023</td><td>Observational study</td><td>Conducted in Germany</td></tr>
</table>

<table>
<tr><th colspan="4">RESEARCH CONTENT</th></tr>
<tr><th>OBJECTIVES</th><th>SAMPLE</th><th>RESULTS</th><th>CONCLUSIONS</th></tr>
<tr>
<td>Evaluation of the efficacy and safety of SG in clinical practice as 2nd-3rd choice agent in Germany (Kliniken Essen-Mitten).

Most patients will receive the standard dose of 10 mg/kg, but some will receive 7.5 mg/kg based on clinical judgment and previous severe AEs.</td>
<td>(n = 48)
Patients with de novo or metastatic TNBC who receive SG as 2nd or 3rd choice treatment in routine clinical practice in Germany.
Standard CT regimens previously received:
- Taxanes: 38 p
- A. Platinum: 27 p
- Gemcitabine: 14 p
- Eribulin: 11 p
- PD1/PDL1 Inh: 18 p
- De novo metastatic TNBC: 8 p (18.6%).

- TNBC with primary diagnosis: 23 p (53.5%).</td>
<td>Efficacy of SG:
Median of PFS: 5 months
Median of OS: 13.1 months
AEs:
<table>
<tr><th></th><th>AE's grade 1-4</th><th>AE's grade >3</th></tr>
<tr><td>Neutropenia</td><td>32.6%</td><td>27.9%</td></tr>
<tr><td>Diarrhea</td><td>37.2%</td><td>18.6%</td></tr>
<tr><td>Febrile Neutropenia</td><td>-</td><td>4.7%</td></tr>
<tr><td>Anemia</td><td>34.9%</td><td>4.7%</td></tr>
<tr><td>nausea</td><td>-</td><td>4.7%</td></tr>
<tr><td>elevated transaminases</td><td>30.2%</td><td>4.7%</td></tr>
<tr><td>fatigue</td><td>34.9%</td><td>2.3%</td></tr>
<tr><td>vomiting</td><td>7%</td><td>2.3%</td></tr>
<tr><td>difficulty breathing</td><td>16.3%</td><td>2.3%</td></tr>
<tr><td>alopecia</td><td>90.7%</td><td>-</td></tr>
</table>
In general, AEs were more frequent and severe in older patients, but these data cannot be interpreted as</td>
<td>The real-world safety and efficacy profile of SG in patients with TNBC is consistent with data from previous clinical trials such as ASCENT. Further studies are needed to optimize the optimal use of SG as monotherapy in TNBC, particularly in the context of appropriate management of associated AEs.</td>
</tr>
</table>

	**- TNBC without primary diagnosis: 22 p**<br>**- HR+ 14 p**<br>**- Her2neu 6 p**<br><br>**Metastases (n = 36):**<br>**-** Lymph: 30 p<br>- Bone: 22 p<br>- Lung: 22 p<br>- Liver: 22 p	clinically significant as the sample size is too small to be confident.<br>• There were 8 deaths (18.6%), but all were due to disease progression and not related to SG toxicities.<br>**Prophylaxis used:**<br>• Alopecia: cold cap (n = 5).<br>○ No benefit was demonstrated.<br>• Neutropenia: Primary prophylaxis G-CSF (n=11)<br>○ Indicated in patients at high risk of developing neutropenia due to a history of neutropenia.<br>• Neutropenia: Secondary Prophylaxis G-CSF (n=4)<br>○ Indicated in patients experiencing grade 3 neutropenia or febrile neutropenia during treatment with SG.<br>• Diarrhea: Secondary Prophylaxis with Loperamide	

<table>
<tr><th colspan="5">PUBLICATION DETAILS</th></tr>
<tr><th>REFERENCE</th><th>AUTHORS</th><th>DATE</th><th>TYPE OF STUDY</th><th>CLINICAL TRIAL</th></tr>
<tr><td>[60]</td><td>Spring LM., et al.</td><td>12.12.2023</td><td>Phase III clinical trial</td><td>NeoSTAR</td></tr>
<tr><th colspan="5">RESEARCH CONTENT</th></tr>
<tr><th>OBJECTIVES</th><th>SAMPLE</th><th colspan="2">RESULTS</th><th>CONCLUSIONS</th></tr>
<tr><td>To analyze complete ORR in the breast and lymph nodes, in addition to PFS, OS, DOR, safety, incidence of AEs and possible predictive biomarkers, in the treatment of SG at the standard dose of 10 mg/kg on days 1 and 8 of 21-day cycles.</td><td>(n = 50)
Patients with localized TNBC with good prognosis and tumor size >1cm with positive lymph node:
- Stage I: 13 p
- Stage II: 26 p
- Stage III: 11 p
- Stage IV: 0 p

BRCA patients:
- Positive: 9 p
- Negative: 40 p</td><td colspan="2">Study Procedures (After 4 cycles, the following was observed)
• Disease progression:
  ○ Discontinue SG and proceed to surgery.
  ○ Receive additional neoadjuvant treatment with CT agents.
• Residual disease in breast and/or axilla (n=14):
  ○ Receive additional neoadjuvant platinum or taxane + surgery.
    ▪ 13 patients
• Complete or near complete response to XR
  ○ Definitive surgery (mastectomy with sentinel node evaluation and possible axillary lymph node dissection).
    ▪ 16 patients
  ○ Received additional adjuvant therapy.
• 20 patients received additional neoadjuvant therapy:
  ○ 7 patients had a pCR and did not receive subsequent adjuvant CT.
  ○ 13 patients did not have a pCR and therefore received subsequent adjuvant CT.
• 1 patient was unable to complete the minimum of 4 cycles due to early progression.
Efficacy of SG in patients with localized TNBC:</td><td>This is the first study of neoadjuvant treatment with an ADC in patients with localized, good prognosis TNBC.
SG demonstrated efficacy as a single agent in monotherapy and feasibility of response-guided escalation/de-escalation.
Further research is needed to determine the optimal duration of SG and combination strategies of neoadjuvant SG with other immunotherapeutic treatments. In addition to a systematic protocol for the management and prevention of AEs according to history and potential risk factors, a systematic protocol for the</td></tr>
</table>

		• **pCR**: 30% (95% CI (18%-45%)) Thus, 15 patients have a 100% SG response.	management and prevention of AEs according to history and potential risk factors is needed.

**pCR at 30%**		
TNBC stage I	13 p	50%<br>CI 95% (21%-79%)
TNBC stage II	26 p	27%<br>CI 95% (12%-48%)
TNBC stage III	11 p	18%<br>CI 95% (2%-52%)
TNBC BRCA +<br>(n = 9)	6 p	66.7%<br>CI 95% (30%-93%)

• **ORR:** 32 patients (64% - CI 95% (77% - 98%)

**ORR**		
TNBC stage I	13 p	54%<br>CI 95% (25%-81%)
TNBC stage II	26 p	69%<br>CI 95% (48%-86%)
TNBC stage III	11 p	64%<br>CI 95% (31%-89%)

• **ORR in patients with/without BRCA:**

	**ORR WITHOUT BRCA (n = 40)**	**ORR WITH BRCA (n = 9)**
Sample	67.5%<br>Ci 95% (51%-81%)	55.6%<br>Ci 95% (21%-86%)
RP/RC	27 patients	5 patients
Stable dis.	12 patients	4 patients

Progressive disease	1 patient	-

- **Median of PFS at 2 years:**

**Median of PFS at 2 years**	
In all patients	95%<br>CI 95% (88%-100%)
In patients with pCR +	100%
In patients with residual disease	92%

In patients with a favorable and positive pCR rate, there was 100% progression-free survival in responders during the first two years after treatment initiation.

AEs:

- Nausea: 82%
- Fatigue: 76
- Alopecia: 76
- Skin Rash: 48
- Neutropenia: 44%.
- UGT1A1 genotyping was not performed.

Expression of Trop2:

- (+1 o +0): 17 patients
- (+3 o +2): 28 patients
- There was no significant difference in the rate of pCR in relation to Trop 2 expression. Thus, the pCR results do not appear to be dependent on the expression level of this biomarker. Regardless of Trop 2 expression, SG will have an effective effect without depending on this aspect. The level of Trop 2 does not predict the effect of SG on this tumor.

<table>
<tr><th colspan="5">PUBLICATION DETAILS</th></tr>
<tr><th>REFERENCE</th><th>AUTHORS</th><th>DATE</th><th>TYPE OF STUDY</th><th>CLINICAL TRIAL</th></tr>
<tr><td>[61]</td><td>Kaylee C, et al.</td><td>01.03.2023</td><td>Retrospective analysis</td><td>Mayo Clinic</td></tr>
<tr><th colspan="5">RESEARCH CONTENT</th></tr>
<tr><th>OBJECTIVES</th><th>SAMPLE</th><th colspan="2">RESULTS</th><th>CONCLUSIONS</th></tr>
<tr><td>Analysis of the Management of Neutropenia Following SG Treatment in Patients with TNBC.</td><td>(n = 67)
Patients with TNBC who have received at least two doses of SG.</td><td colspan="2">Breakdown of G-CSF prophylaxis in patients with TNBC who were treated with SG:
A total of 42 patients (63%) received G-CSF support during SG treatment.
G-CSG support was most commonly added during the first two cycles (59.5%).
PRIMARY PROPHYLAXIS: 12 patients-initiated G-CSF with no history of neutropenia-related delays and no neutropenia at the time of administration (i.e., no indication, but as primary prophylaxis).
Primary prophylaxis in cycle 1: 11 patients.
SECONDARY PROPHYLAXIS: 21 patients (81%) were treated with the addition of G-CSF (13 pegfilgrastim, 8 filgrastim) after neutropenia was reported as an AE of SG treatment.
OS DELAY x NEUTROPENIA: 26 patients (39%) with treatment delay due to neutropenia while receiving SG.
Median number of SG cycles per patient event:<table><tr><td>Median number of cycles for all patients</td><td>5 cycles (1-25)</td></tr><tr><td>Median number of cycles in patients with G-CSF</td><td>5 cycles (1-25)</td></tr><tr><td>Median number of cycles in patients without prophylaxis</td><td>4 cycles (1-19)</td></tr></table></td><td>There is no pre-established or standardized protocol to follow in the presence of neutropenia as an AE of SG treatment in patients with TNBC. Therefore, it is considered an area to be studied in the future in order to understand its management and to be able to offer a treatment with G-CSF according to the needs of each patient.</td></tr>
</table>

<table>
<tr><th colspan="5">PUBLICATION DETAILS</th></tr>
<tr><th>REFERENCE</th><th>AUTHORS</th><th>DATE</th><th>TYPE OF STUDY</th><th>CLINICAL TRIAL</th></tr>
<tr><td>[62]</td><td>Olivier T, et al</td><td>01.2022</td><td>Systematic review</td><td>ASCENT</td></tr>
<tr><th colspan="5">RESEARCH CONTENT</th></tr>
<tr><th>OBJECTIVES</th><th>SAMPLE</th><th colspan="2">RESULTS</th><th>CONCLUSIONS</th></tr>
<tr><td>Analysis of errors and biases in the ASCENT trial methodology that may have obscured the very promising SG results compared to standard CT treatment..</td><td>(n = 529)</td><td colspan="2">• - OPEN LABEL DESIGN (OPEN TRIAL): Both patients and investigators know whether the drug they are receiving is experimental or control (so there is no blinding). Remember that open trials tend to exaggerate the effect of the experimental group. In fact, it is known that in the control group (233 patients), 32 of these patients withdrew their consent and did not start the CT treatment because the randomization process did NOT allow these patients access to the experimental group.
• The ASCENT clinical trial, was EARLY DETERMINED EARLY: This was done on the recommendation of an independent committee due to the very positive evidence of efficacy. This may lead to an overstatement of the magnitude of the benefit according to the statistics (it greatly increases PFS but does not affect SG). The veracity of PFS values may be altered or refuted by
○ SG is much more useful than PFS in patients with lethal malignancies.
○ PFS is a poor predictor of SG in metastatic TNBC.
○ PFS in an open-label model may not accurately capture therapeutic benefit.
○ This means that we should rely more on SG in the ASCENT trial.</td><td>El grupo de control deficiente del ensayo ASCENT impide una respuesta definitiva sobre la eficacia del fármaco experimental en comparación con la atención estándar. Y los pacientes no seleccionados del mundo real no recibirán el fármaco experimental según las reglas del ensayo: no está claro si obtendrán el mismo beneficio. El efecto acumulativo de cada diseño de características descrito en el ensayo ASCENT tiene el potencial de distorsionar los verdaderos resultados de eficacia.</td></tr>
</table>

		• **The CONTROL (CT) arm of the ASCENT trial is DEFICIENT (more sample needed):** Some patients did not receive all possible standard CT therapies (some did not receive platinum (31%) or anthracyclines (17%), and these CTs would have been more effective in these patients).<br>o ANTHRACYCLINES are the main CT in metastatic TNBC and CM in general. So, if 17% were not exposed to this treatment, they could have had better benefits (also is that 17% is a low percentage, but these patients if given anthracyclines instead of another TC during the trial, could have further increased rates of PFS, OS, ORR...).<br>o However, an unexpected improvement was seen in patients who received eribulin, so it could be a new main treatment in TNBC, and so it would have been interesting to test eribulin and anthracyclines in more patients in the control sample.<br>o It is known that there is a better response in patients with BRCA mutation treated with CARBOPLATIN and therefore NCCN recommends this therapy in patients with TNBC + BRCA.<br>• **There were errors and imbalances in the recommendations/protocol for SG dose reductions in the face of severe AEs:**<br>o SG dose modifications in the ASCENT trial are the same as in the FDA label, but during the trial	

			SG patients were not treated according to these rules:<br>o In FEBRILE NEUTROPENIA:<br>▪ SG: After the 1st episode, NO dose reduction was recommended, but G-CSF was prescribed.<br>▪ CT: After the 1st episode, dose reductions were applied, but G-CSF prescription was NOT mandatory**.**	

Abbreviations: TNBC: Triple negative breast cancer. FDA: Food and Drug Administration. ORR: Objective response rate. PR: Partial response. CR: Complete response. CBR: Clinical benefit rate. (m)DOR: Duration of response. (m)PFS: Progression-free survival. (m)OS: Overall Survival. AE(s): Adverse events. SG: Sacituzumab govitecan. CT: Chemotherapy. TDP: Time to disease progression. BM: Brain Metastasis. UGT(1A1): Uridina difosfato glucoronosil transferasa 1A1. G-CSF: Granulocyte colony-stimulating factor. Ab: Antibody. ECOG: Eastern Cooperative Oncology Group. RI: Renal insufficiency. Ag: Antigen. Hb: Hemoglobin. NCCN: National Comprehensive Cancer Network. ADC: Antibody-drug conjugates. E: Eribulin. V: Vinorelbine. C: Capecitabine. G: Gemtacibin. RT: Radiotherapy. ANC: Absolute neutrophil count. AST: Aspartate aminotransferase. ALT: Alanine aminotransferase. NCI: National Cancer Institute. HRQoL: Health-related Quality of Life. QoL: Quality of Life. BB: Bilirubin. MRI: Magnetic Resonance Imaging. CT: Computed tomography. Febrile N.: Febrile Neutropenia. HR: Hormone receptors. CAR: Carboplatin. ATE: Atezolizumab. EMA: European Medicines Agency. XR: Radiography (X-ray). ELISA: Enzyme-Linked ImmunoSorbent Assay. IUNL: Institutional Upper Normal Limit. CrCL: Creatinine Clearance. SI: Symptomatic impact. pCR: pathologic complete response
